# Brain age revisited: Investigating the state vs. trait hypotheses of EEG-derived brain-age dynamics with deep learning

**DOI:** 10.1162/imag_a_00210

**Published:** 2024-07-08

**Authors:** Lukas A.W. Gemein, Robin T. Schirrmeister, Joschka Boedecker, Tonio Ball

**Affiliations:** Neuromedical AI Lab, Department of Neurosurgery, Medical Center – University of Freiburg, Faculty of Medicine, University of Freiburg, Freiburg, Germany; Neurorobotics Lab, Computer Science Department – University of Freiburg, Faculty of Engineering, University of Freiburg, Freiburg, Germany; Machine Learning Lab, Computer Science Department – University of Freiburg, Faculty of Engineering, University of Freiburg, Freiburg, Germany; BrainLinks-BrainTools, Institute for Machine-Brain Interfacing Technology, University of Freiburg, Freiburg, Germany; Freiburg Epilepsy Center, Department of Neurosurgery, Medical Center – University of Freiburg, Faculty of Medicine, University of Freiburg, Freiburg, Germany

**Keywords:** EEG, electroencephalography, brain, age, aging, decoding, deep learning, convolutional neural networks, biomarker, pathology

## Abstract

The brain’s biological age has been considered as a promising candidate for a neurologically significant biomarker. However, recent results based on longitudinal magnetic resonance imaging (MRI) data have raised questions on its interpretation. A central question is whether an increased biological age of the brain is indicative of brain pathology and if changes in brain age correlate with diagnosed pathology (state hypothesis). Alternatively, could the discrepancy in brain age be a stable characteristic unique to each individual (trait hypothesis)? To address this question, we present a comprehensive study on brain aging based on clinical Electroencephalography (EEG), which is complementary to previous MRI-based investigations. We apply a state-of-the-art temporal convolutional network (TCN) to the task of age regression. We train on recordings of the Temple University Hospital EEG Corpus (TUEG) explicitly labeled as non-pathological and evaluate on recordings of subjects with non-pathological as well as pathological recordings, both with examinations at a single point in time TUH Abnormal EEG Corpus (TUAB) and repeated examinations over time. Therefore, we created four novel subsets of TUEG that include subjects with multiple recordings: repeated non-pathological (RNP): all labeled non-pathological; repeated pathological (RP): all labeled pathological; transition non-patholoigical pathological (TNPP): at least one recording labeled non-pathological followed by at least one recording labeled pathological; and transition pathological non-pathological (TPNP): similar to TNPP but with opposing transition (first pathological and then non-pathological). The results show that our TCN reaches state-of-the-art performance in age decoding on non-pathological subjects of TUAB with a mean absolute error of 6.6 years and an$R^{2}$score of 0.73. Our extensive analyses demonstrate that the model underestimates the age of non-pathological and pathological subjects, the latter significantly (-1 and -5 years, paired t-test,p = 0.18andp = 6.6e−3). Furthermore, there exist significant differences in average brain age gap between non-pathological and pathological subjects both with single examinations (TUAB) and repeated examinations (RNP vs. RP) (-4 and -7.48 years, permutation test,p = 1.63e−2andp = 1e−5). We find mixed results regarding the significance of pathology classification based on the brain age gap biomarker. While it is indicative of pathological EEG in datasets TUAB and RNP versus RP (61.12% and 60.80% BACC, permutation test,p = 1.32e−3andp = 1e−5), it is not indicative in TNPP and TPNP (44.74% and 47.79% BACC, permutation test,p = 0.086andp = 0.483). Additionally, all of these classification scores are clearly inferior to the ones obtained from direct EEG pathology classification at 86% BACC and higher. Furthermore, we could not find evidence that a change of EEG pathology status within subjects relates to a significant change in brain age gap in datasets TNPP and TPNP (0.46 and 1.35 years, permutation test,p = 0.825andp = 0.43; and Wilcoxon-Mann-Whitney and Brunner-Munzel test,p = 0.13). Our findings, thus, support the trait rather than the state hypothesis for brain age estimates derived from EEG. In summary, our findings indicate that the neural underpinnings of brain age changes are likely more multifaceted than previously thought, and that taking this into account will benefit the interpretation of empirically observed brain age dynamics.

## 1 Introduction

To describe the speed of aging of people and their individual organ systems, the biological age has been introduced. While the chronological age (CA) is precisely defined by the birth date of a person, the biological age is subject to variation and can diverge from the CA. The biological age can presumably be influenced by environmental factors, by harmful behavior, by acquisition and recovery of diseases and disorders, by cognitive training, and by congenital factors. The divergence of CA and biological age might have clinical importance as it might contribute to a better understanding of healthy or pathological variations, for example, in the context of blood and heart (Pavanello et al., 2020), the skin (Kuklinski et al., 2017), or the brain (Ye et al., 2020).

To estimate the biological age of the brain (BA), a decoder model needs to be fit to data of the brain to begin with. Typically, only data of healthy subjects are included in training and the model is optimized to reduce the error between prediction and CA of the subject. While taking potential model bias into account, the final predictions are then considered to be the BA.

A variety of different terms have emerged to describe the difference of BA to CA, for example, ‘brain age gap’ (Franke et al., 2010), ‘brain age delta’ (Vidal-Pineiro et al., 2021), ‘brain-predicted age difference’ (Cole et al., 2018), and ‘brain age index’ (Sun et al., 2019). For the rest of this manuscript, we will refer to the difference of predicted BA and CA of the subject as brain age gap (BAG).

So far, aging of the brain has primarily been studied based on magnetic resonance imaging (MRI) scans (Cole et al., 2017;Franke and Gaser, 2019;Franke et al., 2010,^2012^,^2013^;Gaser et al., 2013;Löwe et al., 2016;Smith et al., 2019,^2020^;Vidal-Pineiro et al., 2021). The best results were reported byVidal-Pineiro et al. (2021),Smith et al. (2019),Smith et al. (2020), andLeonardsen et al. (2022)who were able to decode the BA with a decoding error of 5, 3.3, 2.9, and 2.5 years mean absolute error (MAE), respectively. For a review of brain aging research based on MRI, please refer toFranke and Gaser (2019).

In another line of research, BA has been studied based on magnetoencephalography (MEG) and electroencephalography (EEG) data (Banville et al., 2023;Bomatter et al., 2023;Bonet et al., 2023;Engemann et al., 2022;Paixao et al., 2020;Sabbagh et al., 2019,^2020^;Sun et al., 2019;Xifra-Porxas et al., 2021;Ye et al., 2020). The best reported scores are 4.88 years MAE for a combination of MEG and MRI data, 6.6 years MAE for MEG data, and 5.96 years MAE for EEG data byXifra-Porxas et al. (2021),Bonet et al. (2023), andKhayretdinova et al. (2022), respectively. Just recently,Engemann et al. (2022)also published a benchmark for brain age decoding from multiple MEG and EEG datasets that can be used for thorough model comparisons.

The review of related literature above indicates that brain age studies based on MRI data generally yield superior decoding results, that is, lower MAE. However, the advantages of EEG over MRI justify continued interest in developing reliable brain age estimation methods using EEG data. MRI is more expensive, requires specialized facilities, and is less comfortable for patients due to the loud noises and confinement. EEG is less expensive, silent during operation, suitable for individuals with claustrophobia, and potentially portable for use in home settings. The availability of EEG systems is also considerably higher than that of MRI systems, due to their lower acquisition and running cost. Therefore, the development of a reliable and precise BA decoder based on EEG signals would be valuable.

Several studies have put the decoded BA and the resulting brain age gap in relation to pathologies.Franke (2013)found an increase in brain age gap in patients with type 2 diabetes mellitus which also increased with longer diabetes duration.Ye et al. (2020)found an increased brain age gap in patients with dementia. Similarly,Sun et al. (2019)presented excess brain age in patients with significant neurological or psychiatric diseases, hypertension, and diabetes.Liem et al. (2017)found advanced brain age in patients with cognitive impairment. Ultimately,Paixao et al. (2020)reported an decreased life expectancy in patients with excess brain age gap. Similarly,Cole et al. (2017)found early mortality in subjects with higher brain age.

In contrast to the works listed above,Vidal-Pineiro et al. (2021)argue that the brain age gap is primarily influenced by congenital factors like birth weight and other polygenic factors.

From the review of related literature above, it appears that two fundamentally different views on the brain age gap exist, which we will refer to as the state and trait hypotheses:

I)  **State hypothesis**: The brain age gap and its trajectory are subject to variation over time and can hence indeed be influenced, for example, by life events, environmental factors, illness, etc.
II)  **Trait hypothesis**: The brain age gap is determined by early-life or genetic factors and follows a predetermined trajectory unaffected by life events, environmental factors, illness, etc.

The two views are reminiscing the concept of trait and state anxiety as widely used in psychology (Spielberger et al., 1971). We propose that the brain age might be conceptualized in a similar way. It might have a trait component (as described in II) that is fixed and predetermined by congenital factors. Additionally, there might be a state component (as described in I) that changes over time, for example with acquisition of a disease. Furthermore, the state hypothesis implies that this component of the BA might also be reversible, that is, by recovery of the disease or by cognitive training. Finally, it is important to note that the two views as described above are not mutually exclusive: There might be both, a state and a trait component in BA.

However, studies testing these two fundamentally different views of the brain age gap against each other are so far scarce and, to the best of our knowledge, there is no study so far addressing this issue based on clinical EEG.

To address this open issue, in this study, we first aimed to improve the performance of BA estimation from EEG signals beyond the current state-of-the-art. To this aim, we adapted an established decoder model from EEG pathology classification. We fit the model to EEG recordings of non-pathological subjects, minimizing the error between predictions and CA of the subjects. Next, we predicted recordings of non-pathological as well as pathological subjects and computed their brain age gap. Then, we analyzed the predictive value of the brain age gap with respect to clinically defined EEG pathology. We further investigated the concepts of state and trait brain age gap by examining both populations with recordings at a single point in time and populations with repeated recordings over time with and without change of EEG pathology status in the meantime based on novel derivatives of the largest currently available open clinical EEG dataset.

## 2 Materials and Methods

### 2.1 Temple University Hospital EEG Corpus

The Temple University Hospital (TUH) EEG Corpus (TUEG) (v1.2.0) (Obeid & Picone, 2016) is the largest publicly available resource of clinical EEG recordings to date, with almost 70,000 recordings from over 15,000 subjects (7664 female, 7321 male, 16 undetermined). Along with recordings in European Data Format (EDF) (Kemp et al., 1992), the dataset included medical reports in plain text, providing additional information, such as medication, anamneses, and EEG findings. By default, TUEG does not offer labels indicating pathological EEG. They were generated according to the routine detailed inKiessner et al. (2023). Similar efforts have been made byWestern et al. (2021). TUEG features multiple predefined subsets, including a Seizure Corpus (Shah et al., 2018), an Epilepsy Corpus (Veloso et al., 2017), a Slowing Corpus (von Weltin et al., 2017), an Artifact Corpus (Buckwalter et al., 2021), and a general Abnormal Corpus (Lopez de Diego, 2017). In this study, we used the TUH Abnormal EEG Corpus to train our age decoder and created four novel subsets of TUEG to study aging effects over time.

#### 2.1.1 Temple University Hospital Abnormal EEG Corpus

The TUH Abnormal EEG Corpus (TUAB) (v2.0.0) (Lopez de Diego, 2017) is a subset of TUEG (Obeid & Picone, 2016) and comprises a total of 2993 recordings (1715 non-pathological/1278 pathological) of 2329 subjects (1255 female / 1074 male). The dataset is divided into a predefined training set (2717 recordings) and a final evaluation (FE) set (276 recordings), which were not aligned in terms of age distribution. To avoid resulting bias when training our decoders, we re-split the dataset. We split the data on a subject-wise basis to prevent patient leak between the training and FE sets (Fig. S5). The dataset contains a mixed bag of pathologies, including, but not limited to, epilepsy, stroke, and Alzheimer’s, lacking easily accessible additional information. In the context of this work, when we refer to EEG pathology, we refer to the binary labels of the TUAB dataset that were created based on EEG assessment of board-certified neurologists (Lopez de Diego, 2017). For the datasets derived from TUEG no such labels exist, such that we created them following the routine described inKiessner et al. (2023).

#### 2.1.2 Novel Temple University Hospital dataset derivatives

To study the effects of aging over time, we created four novel data derivatives from TUEG. First, we excluded recordings from the Seizure, Epilepsy, and Artifact Corpora to avoid misleading EEG activity. We then used pathology labels for the remaining recordings created by mining their medical reports and applying a rule-based text classifier (Kiessner et al., 2023). We selected recordings of at least 2 minutes duration that featured all 21 electrodes of the international 10-20 placement (Jasper, 1958). Finally, we only included patients with at least two recordings.

These datasets include:

I)  **Repeated Non-Pathological (RNP)**:
  This dataset only includes patients with recordings exclusively labeled non-pathological, yielding a total of 4068 recordings of 956 subjects (554 female, 402 male).
II)  **Repeated Pathological (RP)**:
  This dataset only includes patients with recordings labeled pathological, yielding a total of 18,338 recordings of 2892 subjects (1478 female, 1414 male).
III)  **Transition Non-Pathological Pathological (TNPP)**:
  This dataset includes patients with at least one recordings labeled non-pathological and at least one recording labeled pathological, where all pathological recordings chronologically follow the non-pathological recordings. This selection yielded 914 recordings (438 non-pathological, 476 pathological) of 195 subjects (104 female, 91 male).
IV)  **Transition Pathological Non-Pathological (TPNP)**:
  This dataset includes patients with at least one recorded labeled non-pathological and at least one recording labeled pathological, where all non-pathological recordings chronologically follow the pathological recordings. This selection yielded 1273 recordings (686 non-pathological, 587 pathological) of 242 subjects (123 female, 119 male).

#### 2.1.3 Inclusion and exclusion criteria

For the novel datasets RNP, RP, TNPP, and TPNP, we introduced a minimum recording duration of 15 minutes to better align with the TUAB dataset. Note that rejecting individual recordings can trigger the exclusion of additional recordings. For example, if a subject was included with two recordings and one was rejected, it would cause the exclusion of the second recording as well, since the subject no longer qualifies for analysis over time.

As the TUEG dataset offers multiple possibilities to retrieve age information, we compared the extracted age values (Supplement Section A.1). As the comparison revealed substantial differences for some recordings, we additionally implemented an exclusion criterion based on age. We rejected recordings where ages extracted from the different sources showed a deviation of more than 1 year (which can occur naturally due to anonymization). The final selection resulted in 2647 recordings from 2159 subjects for TUAB, 621 recordings from 245 subjects for RNP, 3963 recordings from 1321 subjects for RP, 330 recordings from 133 subjects for TNPP, and 347 recordings from 136 subjects for TPNP. Patient and recording numbers of the data derivatives as well as effects of the inclusion and exclusion criteria are detailed inSupplement Section A.2.

### 2.2 Decoding pipeline

#### 2.2.1 Preprocessing

We applied simple preprocessing steps established in our previous studies (Gemein et al., 2020;Kiessner et al., 2023;Schirrmeister, Gemein, et al., 2017). We picked 21 channels according to the international 10-20 system and clipped extreme technical outlier values to±800μV. We re-referenced all recordings to common average referencing and resampled the signals to 100 Hz. As we have observed a higher number of outliers in the first minute of recordings, we dropped it (Supplement Section A.3). These preprocessing steps were applied to all datasets used in this study, including TUAB, RNP, RP, TNPP, and TPNP. Preprocessing was performed through braindecode^1^(Gramfort et al., 2013;Schirrmeister, Springenberg, et al., 2017) and relies on the MNE^2^library (Gramfort et al., 2014).

#### 2.2.2 Model

We used a temporal convolutional network (TCN) (Bai et al., 2018) as implemented in the Braindecode (BD) library which we refer to as BD-TCN. This model has previously been successfully applied in our pathology decoding study (Gemein et al., 2020), where it achieved superior results. However, in this study, we used the BD-TCN in a regression setting, as opposed to the classification setting used previously. To accomplish this, we replaced the final softmax layer by a sigmoid layer. The hyperparameters of the model (Table S3) were optimized to decode age based on TUAB in a previous Masters Thesis (Chrabąszcz, 2018). For more information about the model architecture, please refer toBai et al. (2018).

#### 2.2.3 Training

We trained our model to decode age of non-pathological recordings of TUAB only. This yielded superior results compared to including recordings that contain pathological activity during training, as observed in preliminary experiments. To facilitate model training, we standardized the recordings channel-wise to zero mean and unit variance by computing the mean and standard deviation of all channels across all recordings in the training split. Additionally, we min-max scaled targets to [0, 1] with respect to ages in the training split of the data.

We employed a training technique known as “cropped decoding” (Schirrmeister, Springenberg, et al., 2017) which produces maximally overlapping data crops through a sliding window approach by shifting one sample at a time. To artificially increase the amount of training data and enhance generalizability, we applied channel dropout data augmentation (Saeed et al., 2021), which emerged as the most promising technique from preliminary experiments.

As in previous studies (Gemein et al., 2020;Kiessner et al., 2023;Schirrmeister, Gemein, et al., 2017), we used the AdamW optimizer (Loshchilov & Hutter, 2017) with a cosine annealing learning rate scheduling (Loshchilov & Hutter, 2016) to optimize the parameters of the network. We trained our network for 35 epochs with a batch size of 128, which was was sufficient to reach convergence of the L1 loss (also known as MAE loss).

The model and its training were implemented using the braindecode library (Gramfort et al., 2013;Schirrmeister, Springenberg, et al., 2017) which relies on Skorch^3^(Tietz et al., 2017), that implements the well-known scikit-learn^4^(Pedregosa et al., 2011) API for artificial neural networks, and PyTorch^5^(Paszke et al., 2017).

#### 2.2.4 Evaluation

We followed the same approach as in our previous study (Gemein et al., 2020) and performed five-fold cross-validation (CV) on the training data of TUAB with the same fixed seed. This ensured that every recording was predicted as part of the validation set exactly once (Fig. S6). In addition to this, we also predicted the pathological recordings of TUAB (which were not included in the training data) for comparison purposes.

To avoid leakage of recordings of one subject into different subsets, we performed subject-wise splitting of the data and shuffled beforehand. Through CV, we were able to optimize our choices of data augmentation technique and loss function.

After completing CV, we conducted five repetitions of final evaluation (FE) using varying seeds to average out effects of random weight initialization. During FE, we trained our model on all available training data and predicted the held-out final evaluation data.

### 2.3 Metrics

We report all age regression results as the average over five CV runs or as the average over five repetitions of FE using the mean absolute error(MAE):$\sum_{i\;=\;1}^{n}|{\hat{y}}_{i}-y_{i}|/n$

Additionally, we also report the$R^{2}$score, which is frequently used in the literature:$\sum_{i\;=\;1}^{n}{\left({\hat{y}}_{i}-\bar{y}\right)}^{2}/\sum_{i\;=\;1}^{n}{\left(y_{i}-\bar{y}\right)}^{2}.$

In both cases,nis the total number of examples,yis the decoding target,$\hat{y}$is the prediction, and$\bar{y}=\sum_{i\;=\;1}^{n}y_{i}/n$.

Furthermore, we report all classification results as the balanced accuracy (BACC) score:1/2  *​(TP / (TP + FN) +(TN / (TN + FP))), where

TP is the number of examples that were correctly classified as the positive class (pathological),

TN is the number of examples that were correctly classified as the negative class (non-pathological),

FP is the number of examples that were incorrectly classified as the positive class (pathological), and

FN is the number of examples that were incorrectly classified as the negative class (non-pathological)

### 2.4 Post-hoc analyses

#### 2.4.1 Brain age prediction bias

We computed the model bias with respect to the original age distribution by fitting a quadratic regression model to decoding targets and brain age gap (BA-CA) during CV (Fig. S11). Directly after prediction and prior to computing any scores or conducting any further analyses, we applied this model to remove bias from predictions. As a result, all of the results presented in our study were calculated after this correcting step.

This step is necessary as there are direct interactions between the decoding target (chronological age) and the EEG brain age gap (which represents both the decoding error of the model and the expected difference between the brain age and chronological age). This is often neglected in the literature, but it is important to address in order to obtain accurate results. For more information about this issue, please refer toSmith et al. (2019).

#### 2.4.2 EEG brain age gap

We calculated the EEG brain age gap as the difference between the decoded brain age and the chronological age. A positive gap indicates an overestimation of the chronological age, while a negative gap indicates an underestimation of the chronological age. To account for multiple recordings per subject (see recording and patient numbers inSection 2.1.3), we averaged the brain age gaps for each subject. To analyze the difference between the average gaps, we conducted a permutation test.

#### 2.4.3 EEG brain age gap pathology biomarker

We analyzed the predictive value of the EEG brain age gap with respect to EEG pathology. Under the assumption that large deviations from the chronological age may indicate pathological brain activity, we computed two brain age gap thresholds. Gaps that fell within these thresholds were assigned a non-pathological label, while gaps outside of this range were assigned a pathological label.

We selected this combination of thresholds that achieved the highest BACC between the assigned labels and the real pathology labels (as provided by TUAB or as retrieved from medical reports for the datasets with repeated examinations, seeKiessner et al. (2023)). The BACC scores were averaged over the five CV folds.

After completing FE, we applied the thresholds computed during CV and averaged the resulting BACC scores over the five runs. To assess whether the EEG brain age gap was a reliable predictor of EEG pathology, we conducted a randomized test.

#### 2.4.4 Aging effects over time

We computed the change of brain age gap over time. Therefore, we grouped the predictions of our novel datasets by subject and pathology status. If multiple recordings of a subject were performed on the same day, we averaged them. We computed the gap difference of subsequent chronological recordings and divided the brain age gap differences by the time passed in between (so, in other words, the slope of the brain age gap changes over time). We averaged the gap change rates subject-wise and compared the resulting distributions of RNP vs. RP with a Kolmogorov-Smirnov (KS) test, a Wilcoxon–Mann–Whitney test, and a Brunner-Munzel test.

For TNPP and TPNP we also computed the brain age gap rates but instead of averaging we selected the “moment of transition” for each subject, that is, when the pathology label changes between two subsequent recordings. Due to the design of the datasets, this happens exactly once per subject. We then compared the resulting distributions with a KS test, Wilcoxon-Mann-Whitney test, and a Brunner-Munzel test.

#### 2.4.5 Subgroup analysis

We mined medical text reports of RNP and RP to find subjects with conditions Schizophrenia, depression, and stroke. We included a subject in the selection if at least one of its medical reports contained the keyword ‘schizo’ for condition Schizophrenia, ‘depress’ for condition depression, and ‘stroke’ for condition stroke. Capitalization was ignored in all cases. The keyword search yielded 8 subjects in RNP and 33 in RP for Schizophrenia, 20 and 56 subjects for depression, and 15 and 248 subjects for stroke. Review of individual examples confirmed the assumed ways of usage of the keywords, that is, that the subject suffered from the respective condition. Attempted similar stratification of the TNPP and TPNP datasets yielded too small numbers for meaningful analysis.

#### 2.4.6 Statistical testing

We used a paired t-test (Student, 1908) to assess potential differences in the means of the distributions of BA versus CA for both non-pathological and pathological subjects of TUAB.

We used a permutation test with 100,000 samples to assess potential differences in the means of the distributions of brain age gaps of non-pathological versus pathological subjects in TUAB. Furthermore, we assessed whether pathology classification based on the brain age gap biomarker is significantly better than random label assignment in all datasets. We rejected the null hypotheses atp<0.05.

Furthermore, we used a KS test to check for equality of underlying distributions of brain age change rates and a Wilcoxon-Mann-Whitney (WMW) test (Mann & Whitney, 1947;Wilcoxon, 1945) and a Brunner-Munzel test (Brunner & Munzel, 2000) to assess whether brain age gaps in subjects of RNP change at an equal rate over time compared to subjects of RP. Again, we rejected the null hypotheses atp<0.05. More details about the conducted statistical tests can be found inFigure S7.

#### 2.4.7 Amplitude gradient analysis

Following FE, we calculated the gradients of the five models with respect to the non-pathological and pathological recordings of the final evaluation set of TUAB. The gradients were then grouped according to pathology status and subject, and subsequently averaged over all five runs. To visually present which brain regions were most informative for the models in making brain age predictions, we plotted the resulting values on a diagram of the head. The resulting patterns show areas most informative or interesting regarding the brain age decoding task. The bigger the absolute value of the gradients, the more sensitive the network to changes in the input signal with respect to the output (age prediction).

#### 2.4.8 M/EEG brain age benchmark

To evaluate the robustness of our decoding model and to compare it to other established methods, we participated in the recent brain age decoding benchmark (see Engemann et al., 2022 ). This benchmark includes several well-known decoding strategies, ranging from traditional feature extraction with a random forest classifier and computation of covariance matrices with classification based on Riemannian geometry to shallow (BD-Shallow) and deep (BD-Deep) convolutional neural network (CNN) architectures ( Schirrmeister, Springenberg, et al., 2017 ). The benchmark comprises four datasets that vary in ethnicity, demographics, and data type:

I)  TUAB (Lopez de Diego, 2017), recorded from a predominantly North American population,
II)  Leipzig Mind-Brain-Body (LEMON) (Babayan et al., 2019), recorded from a European population with a bimodal age distribution,
III)  Cuban Human Brain Project (CHBP) (Bosch-Bayard et al., 2020;Hernandez-Gonzalez et al., 2011;Valdes-Sosa et al., 2021), recorded from a Latin American population, and
IV)  Cambridge Centre for Ageing and Neuroscience (Cam-CAN) (Shafto et al., 2014;Taylor et al., 2017), the only MEG dataset.

Further details about the datasets and their demographics can be found inEngemann et al. (2022). Our decoding pipeline differs from that presented in the M/EEG brain age benchmarks in several aspects including data format (EDF vs. BIDS (Pernet et al. 2019)) and preprocessing (minimal vs. autoreject (Jas et al. 2017)). An overview and rationale for these differences are provided inTable S2.

## 3 Results

### 3.1 Descriptive statistics

#### 3.1.1 Age distributions

InFigure 1, we present age distributions for RNP, RP, TNPP, and TPNP. The average age is higher in RP, which can be attributed to the increased prevalence of degenerative neurological diseases with advancing age (Hou et al., 2019). Whereas there is a balanced ratio of male to female subjects in RP, there is a moderately higher ratio of female subjects in TNPP and TPNP and a considerably higher ratio of female subjects (60%) in RNP. The variance of ages is lowest for RNP and highest for TNPP. In both TNPP and TPNP, there is a higher number of pathological subjects independent of gender.

**Fig. 1.**
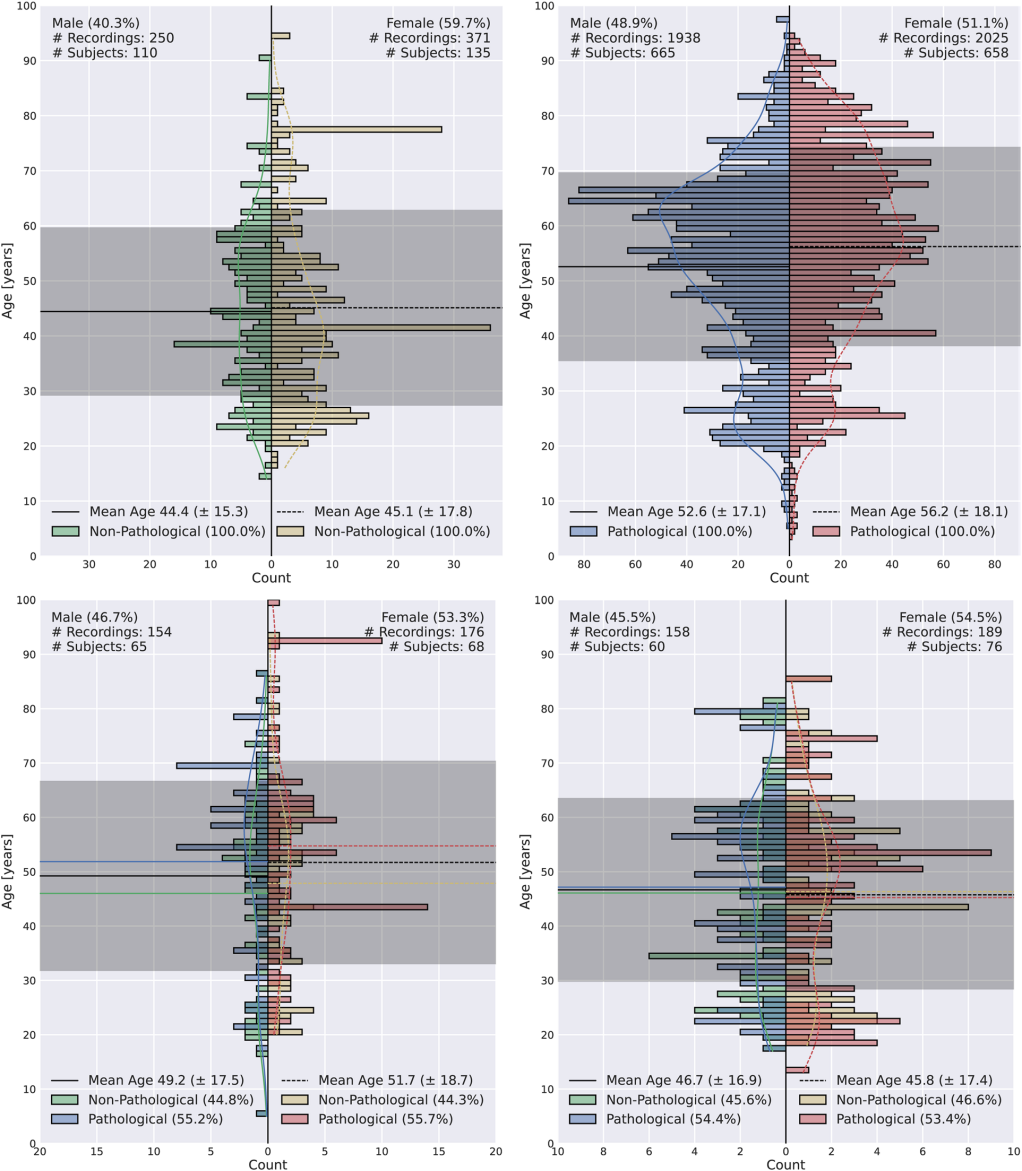
Age distributions of recordings of RNP (top left), RP (top right), TNPP (bottom left), and TPNP (bottom right). Differences in age distribution can be observed independent of dataset and gender. Whereas the distribution of male subjects in RP is bimodal, it is rather uniform in RNP. Average ages of recordings in RP are considerably higher compared to the other datasets. Where average female age is larger than average male age in RNP, RP, and TNPP, this trend is inverted in TPNP. There is a considerably higher percentage of female recordings in RNP compared to RP.

#### 3.1.2 Recordings per subject and intervals

We present the number of recordings per subject in the datasets with repeated examinations in the top panel ofFigure 2. Whereas subjects of RNP, TNPP, and TPNP have a similar number of EEG assessments (approximately 2.5) on average, subjects of RP have three EEG assessments on average. Variance is lowest for TPNP and highest for RP.

**Fig. 2.**
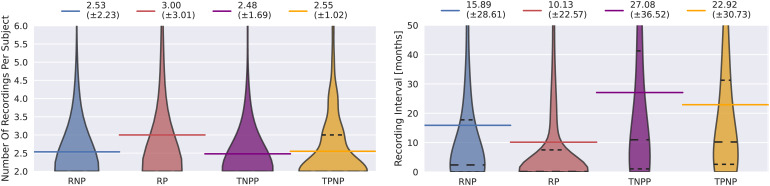
Number of recordings per subject (left) and recording intervals (right) in RNP, RP, TNPP, and TPNP. Colored horizontal lines represent the mean. In RP, there is a higher number of recordings per subject on average compared to all other datasets. There are substantial time gaps of at least 10 months on average between individual recordings in all datasets.

We present the intervals between subsequent recordings of subjects in the novel datasets in the top panel ofFigure 2. On average, there are more than 15 months between recordings of RNP, roughly 10 months between recordings of RP, more than 27 months between recordings of TNPP, and more than 22 months between recordings of TPNP. Variance is lowest in RP and highest in TNPP.

In RNP, more than one recording per subject suggests that these individuals may not be entirely healthy. It is possible that they presented with a condition for which they sought medical attention and underwent EEG analysis, but no signs of EEG pathology were detected despite being ill. In contrast, in RP, TNPP, and TPNP, multiple recordings per subject are reasonable as follow-up analyses may be performed to monitor disease progression or to prevent relapse.

As there are several months between subsequent recordings in all datasets, we assume that observed label transitions correspond to real changes in EEG health status and that non-pathological subjects were not excessively misclassified.

Additional descriptive statistics, including recording durations in the novel derivatives, a more detailed presentation of the non-pathological (NP) population of TUAB, and age distributions of the individual CV folds, can be found inSupplement Section A.5.

### 3.2 Finding 1: TCN reaches state-of-the-art performance on non-pathological subjects of TUAB

**Fig. 3.**
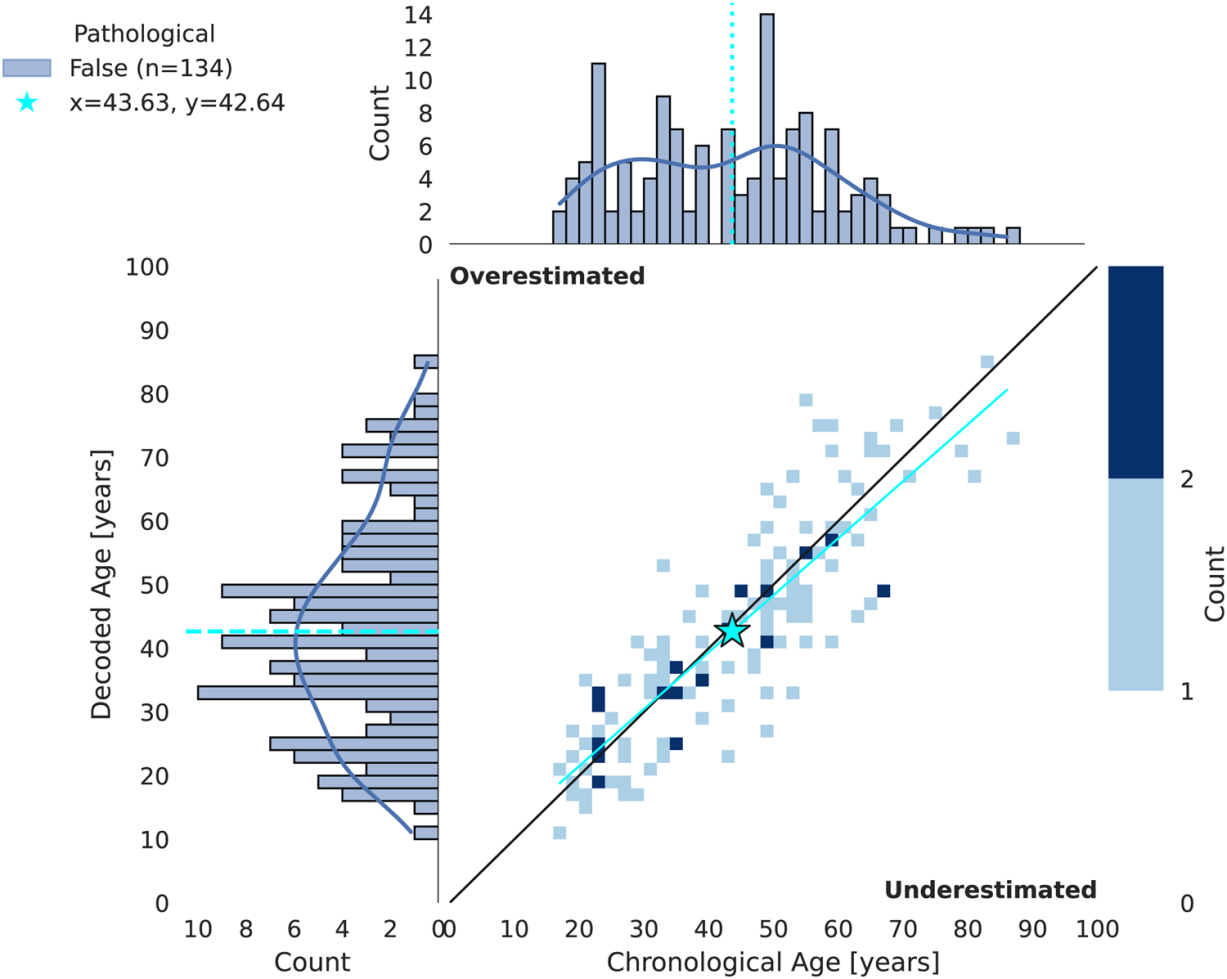
2D histogram of chronological versus decoded age in FE. Individual points represent individual subjects. The decoding models tend to overestimate younger subjects and to underestimate older subjects (cyan trend line). The chronological age is underestimated by 1 year on average (cyan star).

Our brain age decoders achieved an average MAE of 6.60 years and an$R^{2}$score of 0.73 on non-pathological subjects of TUAB during FE.Figure 3presents the decoded BA in relation to CA. Hyperparameters and learning curves of the FE runs demonstrating stable learning and model convergence can be found inSupplement Section A.6 and A.8. CV results can be found inSupplement Section A.7. All regression and classification results as well as statistical analyses at a glance can be retrieved fromSupplement Sections A9, A10, and A11.

To the best of our knowledge, our model achieved state-of-the-art performance. The previous best reported score on TUAB was 7.75 years MAE byEngemann et al. (2022). The overall best score on EEG data is 5.96 years MAE and 0.81 r2 score byKhayretdinova et al. (2022)on the Two Decades-Brainclinics Research Archive for Insights in Neurophysiology (TDBRAIN) database (van Dijk et al., 2022). Our score matches the one presented on MEG data inBonet et al. (2023)(6.6 years MAE), and outperforms the one reported byLeonardsen et al. (2022).

#### 3.2.1 TCN insignificantly underestimates the CA of non-pathological subjects of TUAB

**Fig. 4.**
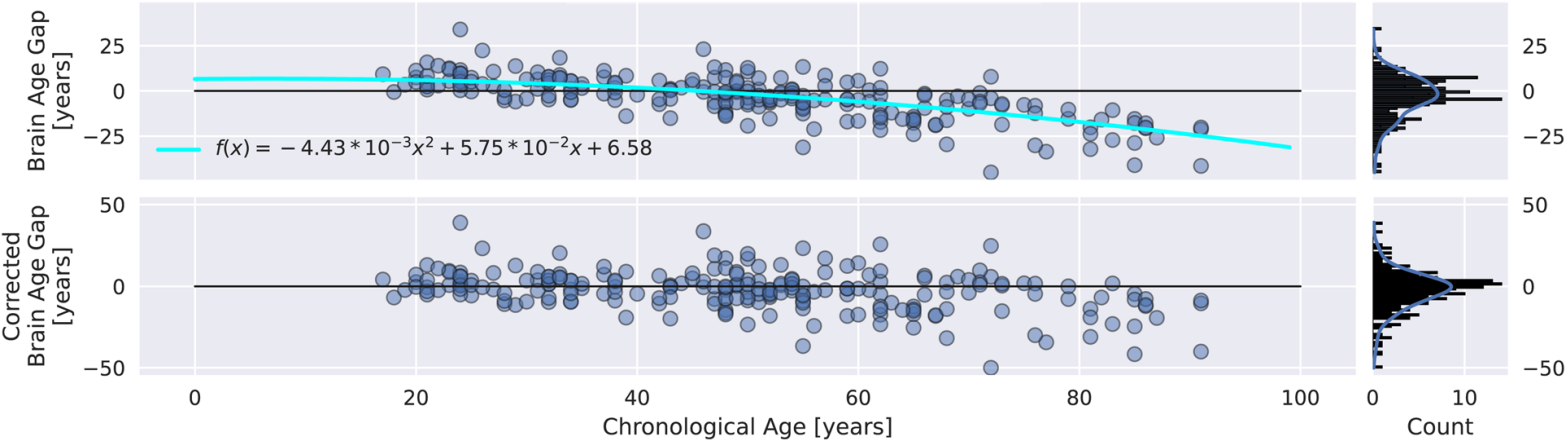
Model bias in FE. (Top) Chronological age versus biased brain age gap and its distribution. (Bottom) Chronological age versus corrected brain age gap and its distribution. Individual points represent individual subjects. The cyan line represents a quadratic bias model fit to CV data. The original distribution of brain age gaps is positively skewed and not centered around zero. Application of the bias model yields the corrected brain age gaps whose distribution is symmetrically centered around zero.

Despite removal of model bias (Fig. 4), we observe an overestimation of CA of younger subjects and an underestimation of CA of older subjects. On average, CA is underestimated by 1 year, which is not statistically significant (paired t-test,p = 0.18).

As explained inSection 2.4.1, removing this bias is essential for robust follow-up analyses. The brain age gap distribution in the top panel ofFigure 4is skewed and not centered around zero. By applying the bias model fit during CV, we were able to account for this bias and to obtain a symmetric distribution centered around zero (bottom panel ofFig. 4). In CV, we observed a similar and stronger effect (Fig. S11).

The removal of the model bias resulted in a minor decrease in raw decoding performance, from 6.48 years MAE (6.51 in CV) to 6.60 years MAE (6.65 in CV). Since information on such a routine is lacking for related works, the resulting reduction in performance could place our work at a disadvantage when compared to decoding results of other studies.

#### 3.2.2 TCN outperforms several competitors on multiple datasets

To demonstrate the robustness of our model, we present it in comparison to other established models and on different datasets through the M/EEG brain age benchmark (Engemann et al., 2022) inFigure 5. It can be seen that our TCN achieves the best score on CHBP, LEMON, and TUAB. On Cam-CAN it achieves results competitive to the other models, out of which Filterbank-Riemann performs best.

**Fig. 5.**
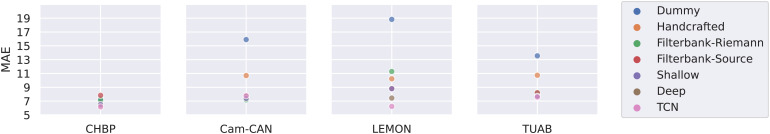
Performance overview and comparison on different datasets through the M/EEG brain age benchmark. The TCN presented in this work reaches top performance in CHBP, LEMON, and TUAB. On Cam-CAN it reaches a slightly lower performance compared to the Filterbank-Riemann approach.

Since our TCN was specifically designed and optimized to decode age from the EEG signals in TUAB, this could be a reason for slightly worse performance on the Cam-CAN dataset. Cam-CAN is the only MEG dataset under investigation that additionally has a larger sensor space compared to the other datasets (Cam-CAN: 102, LEMON: 61, CHBP: 53, TUAB: 21). It is possible that our architecture would require an update or fine tuning to improve performance on this dataset. Our model still shows very solid and robust performance across all four datasets and partially outperforms established decoding methods, which underlines its general applicability to age decoding tasks. Details about differences in our decoding pipeline compared to the one in the M/EEG brain age benchmark can be found inSupplement Section A.4.

### 3.3 Finding 2: TCN significantly underestimates the CA of pathological subjects of TUAB

**Fig. 6.**
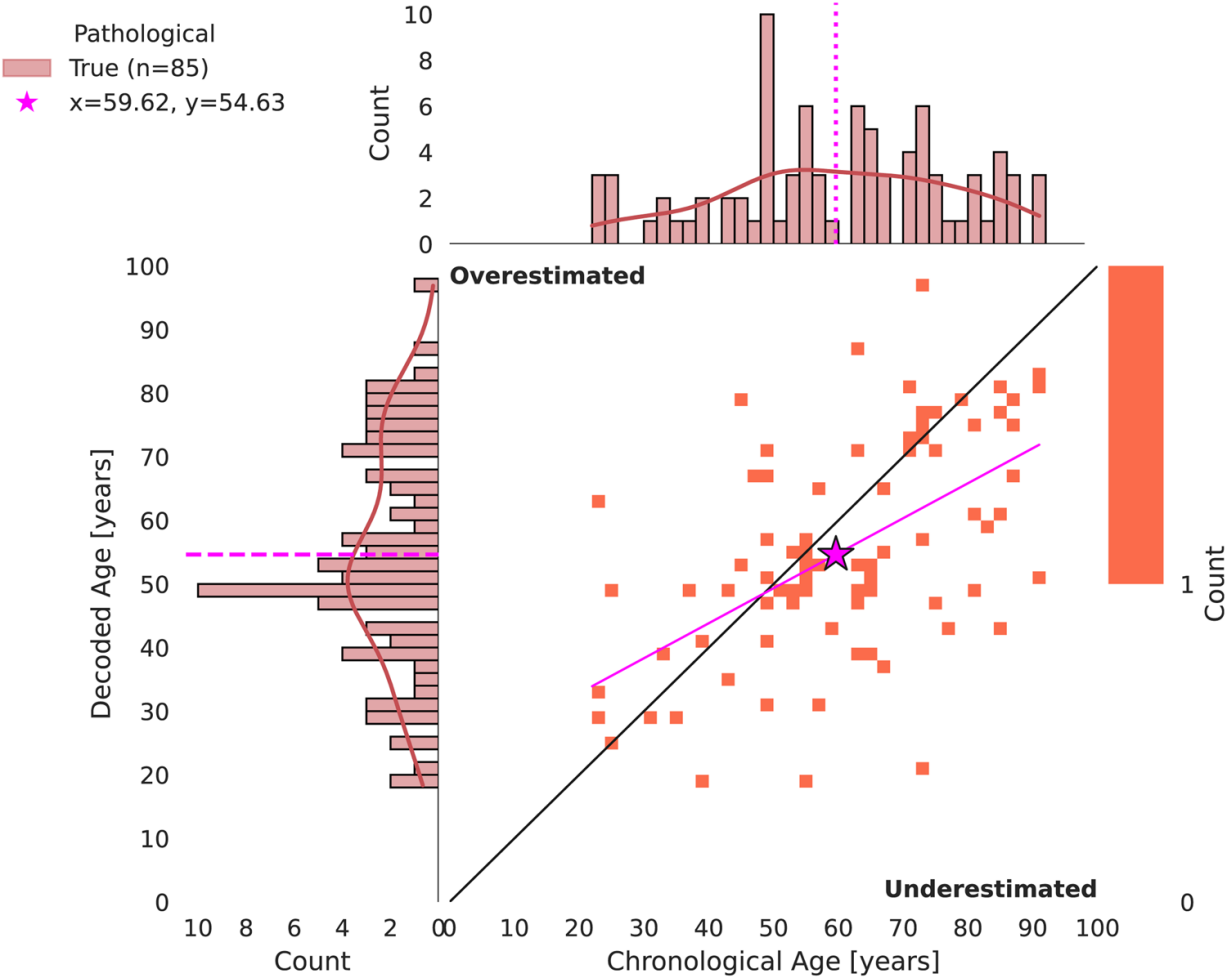
2D histogram of chronological versus decoded age in FE. Individual points represent individual subjects. The decoding models tend to overestimate younger subjects and to underestimate older subjects (magenta trend line). The chronological age is underestimated by 5 years on average (magenta star).

Figure 6shows decoded BA in relation to CA as well as their distributions of pathological subjects of TUAB. Despite removal of model bias beforehand, we again observe an overestimation of younger subjects and an underestimation of older subjects. Overall, ages are underestimated by 5 years on average, which is statistically significant (paired t-test,p = 6.6e-3). Our models reached 12.85 years MAE.

Several aspects become apparent, when comparing the results on non-pathological subjects ( Fig. 3 ) to results on pathological subjects ( Fig. 6 ):

I)  The score on non-pathological subjects is substantially better compared to the score on pathological subjects (6.6 years MAE vs. 12.85 years MAE).
II)  The brain age predictions on non-pathological subjects experience substantially less variance compared to pathological subjects (compare cyan and magenta trend lines and spread of red and blue points).
III)  The brain age predictions on non-pathological subjects experience substantially less bias compared to pathological subjects (compare cyan and magenta star markers).

The presented results are in disagreement with the state hypothesis, since we do not observe an increased BA in pathological subjects. On the contrary, we observe an underestimation of the pathological population overall that is even stronger compared to the non-pathological population. Regarding the trait hypothesis, no clear statement can be made as it does not specify the direction of the brain age gap. There might also be a small preference for increased brain age in pathological subjects, due to neurological diseases and disorders that are induced by genetic factors.

#### 3.3.1 TCN predicts lower BA for pathological than for non-pathological subjects in TUAB

**Fig. 7.**
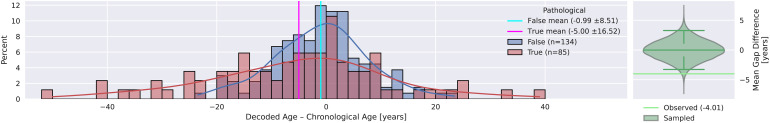
Comparison of brain age gaps of non-pathological and pathological subjects in TUAB through a histogram (left) and a permutation test of average gap differences (right). The cyan and magenta lines represent the average brain age gap of the non-pathological and pathological cohort. The light green line represents the actual observed difference in brain age gap between non-pathological and pathological whereas the green violin represents the distribution sampled as part of a permutation test. Indicators of distribution median, 2.5th, and 97.5th percentile are shown in darker green. Average brain age gaps are negative, while the one of the pathological cohort is smaller. The variance of brain age gaps in the pathological cohort is about twice as high compared to the non-pathological cohort. The difference of average gaps (-4.01) between the cohorts is statistically significant (permutation test,p = 1.63e-2).

Figure 7shows a comparison of brain age gaps of non-pathological versus pathological subjects of TUAB, so basically a comparison of Findings 1 and 2. We find statistically significant differences (permutation test,p = 1.63e-2) in average brain age gaps of non-pathological compared to pathological subjects (Fig. 7to the right).

This finding supports and confirms the previous one: BA of pathological subjects is not only underestimated regarding the CA, but also the underestimation is significantly stronger than the one of non-pathological subjects. Again, this is not in agreement with the state hypothesis while it can be consistent with the trait hypothesis.

### 3.4 Finding 3: pathology classification based on the brain age gap biomarker is vastly inferior to direct pathology decoding

#### 3.4.1 Brain age gap biomarker is less indicative of pathology compared to subject age in TUAB

The top panel ofFigure 8presents the brain age gap as a biomarker of EEG pathology which resulted in a BACC significantly better than chance level (permutation test,p = 1.32e-3). The bottom panel ofFigure 8, on the contrary, shows an analysis of subject age as indicator of EEG pathology as a simple baseline for comparison which yielded a BACC significantly better than chance level (permutation test,p = 1e-5).

**Fig. 8.**
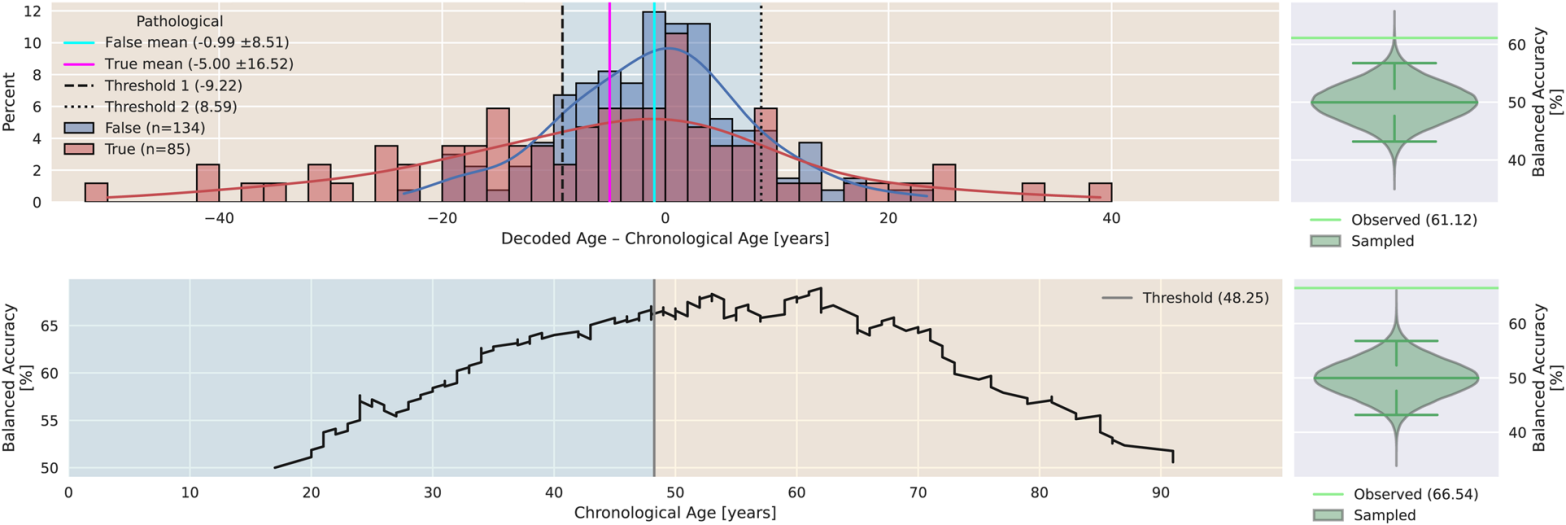
(Top) Comparison of brain age gaps of non-pathological and pathological subjects in TUAB through a histogram (left) with permutation test of pathology proxy analysis (right). The cyan and magenta lines represent the average brain age gap of non-pathological and pathological cohort. The light green line represents the actual observed difference in brain age gap between non-pathological and pathological whereas the green violin represents the distribution sampled as part of a permutation test. Indicators of distribution median, 2.5^th^, and 97.5th percentile are shown in darker green. The biomarker classification yielded a BACC significantly better than chance level (permutation test,p = 1.32e-3). (Bottom) Age threshold pathology proxy in TUAB (left) with permutation test of proxy score (right). Again, blue background signifies non-pathological classification and orange background signifies pathological classification with respect to the age threshold found in CV. The age classification yielded a BACC which is significantly better than chance (permutation testp = 1e-5).

Again, the presented finding is not in agreement with the state hypothesis but can be consistent with the trait hypothesis. If one observed a substantially higher brain age in pathological subjects, then, conversely, this implies that the brain age gap should also be indicative of EEG pathology. Again, the observations can be consistent with the trait hypothesis. As one can achieve a better EEG pathology proxy score by simply exploiting the differences in age distributions between non-pathological and pathological subjects, we conclude that a classification of EEG pathology based on the brain age gap in its presented form is not promising.

#### 3.4.2 Pathology classification based on the brain age gap biomarker is vastly inferior to direct pathology decoding in RNP vs. RP

**Fig. 9.**
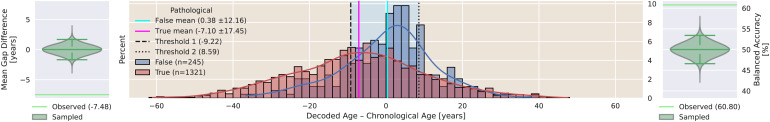
Comparison of brain age gaps of non-pathological and pathological subjects in RNP versus RP (center) with permutation tests of average gap differences (left) and pathology proxy analysis (right). The cyan and magenta lines represent the average brain age gap of non-pathological and pathological cohort. Blue background signifies non-pathological classification and orange background signifies pathological classification based on thresholds found in CV presented as dashed lines. The light green line represents the actual observed difference in brain age gap between non-pathological and pathological, whereas the green violin represents the distribution sampled as part of a permutation test. Indicators of distribution median, 2.5th, and 97.5th percentile are shown in darker green. There is a statistically significant difference (permutation test,p = 1e-5) in average brain age gaps of subjects in RNP versus RP, and the brain age gap biomarker pathology classification reached a BACC statistically significantly better than chance level (permutation test,p = 1e-5).

Figure 9presents brain age gaps of non-pathological versus pathological subjects in novel datasets RNP versus RP, whose average gaps are statistically significantly different (permutation test,p = 1e-5), while the brain age gap biomarker is indicative of pathology (permutation test,p = 1e-5). For a comparison of BA and CA, please refer toFigure S13.

Again, the presented finding is not in agreement with the state hypothesis but can be consistent with the trait hypothesis. The finding demonstrates reproducibility of the previous finding on TUAB on novel datasets with a larger population and repeated consistent pathology assessment.

### 3.5 Finding 4: disease-specific analyses confirm trends observed in the general population

InFigure 10, we present post-hoc analyses of multiple specific conditions, that is, schizophrenia, depression, and stroke. They show trends similar to those of the general populations presented inFigure 9, that is, an underestimation of the brain age extracted from EEG recordings labeled as pathological compared to those labeled non-pathological (permutation test, Schizophrenia:p = 0.4, depression:p = 0.016, stroke:p = 1e-5). While pathology classification based on the brain age gap proxy does not yield a balanced accuracy significantly better than chance in condition depression (permutation test,p = 0.332), it does yield a balanced accuracy significantly better than chance in conditions Schizophrenia and stroke (permutation test,p = 5.67e-3,p = 0.022).

**Fig. 10.**
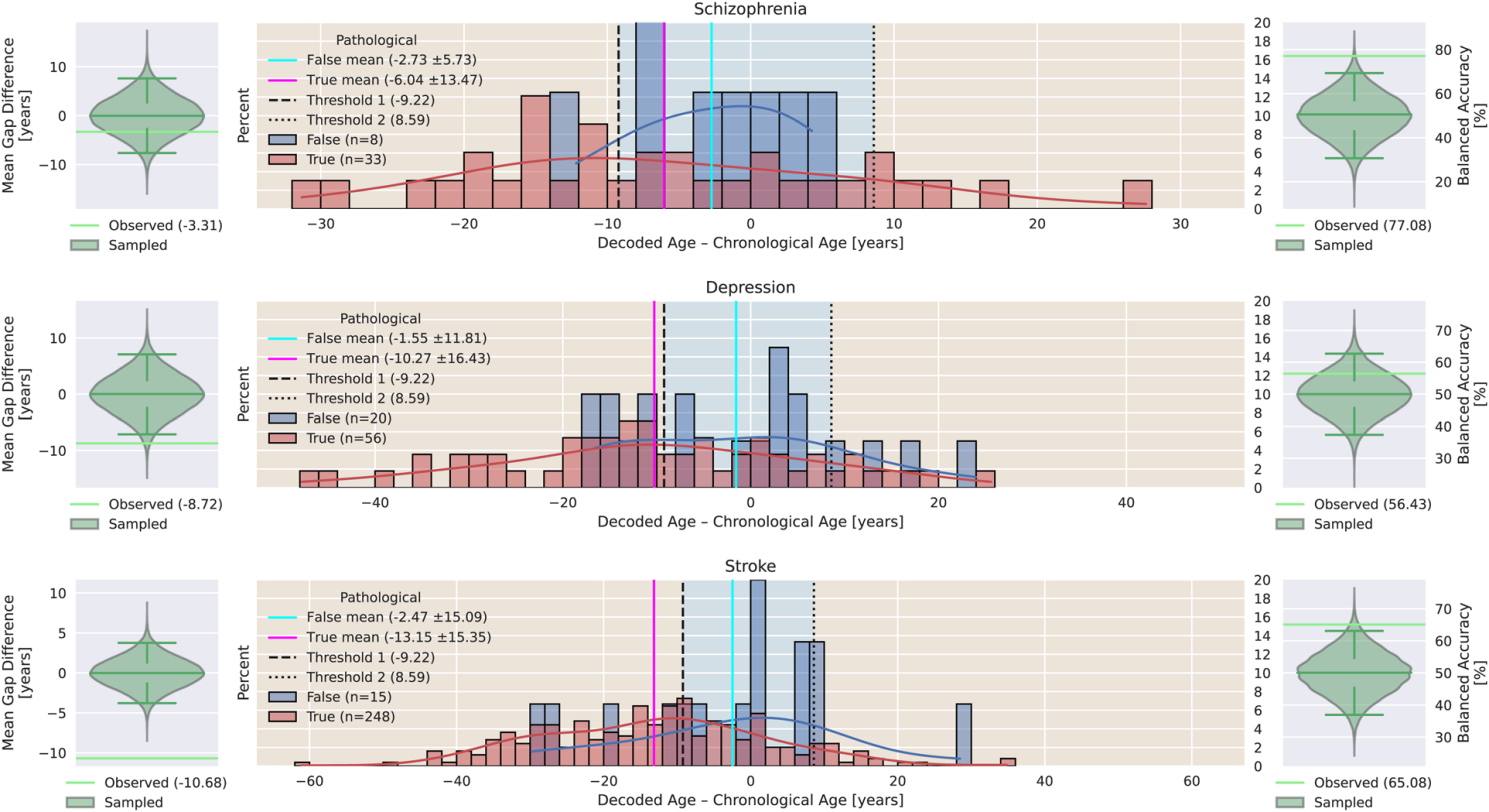
Comparison of brain age gaps of subjects suffering from Schizophrenia (top), depression (center), and stroke (bottom) in RNP and RP with permutation tests of average gap differences (left) and pathology proxy analysis (right). The cyan and magenta lines represent the average brain age gap of non-pathological and pathological cohort. Blue background signifies non-pathological classification and orange background signifies pathological classification based on thresholds found in CV presented as dashed lines. The light green line represents the actual observed difference in brain age gap between non-pathological and pathological whereas the green violin represents the distribution sampled as part of a permutation test. Indicators of distribution median, 2.5th, and 97.5th percentile are shown in darker green. The differences of average non-pathological and pathological gaps are not significant in patients suffering from Schizophrenia, while they are significant in patients suffering from depression and stroke (permutation test, Schizophrenia:p = 0.4, depression:p = 0.016, stroke:p = 1e-5). The application of the pathology proxy thresholds found in CV does not yield BACC scores significantly better than chance level in patients suffering from depression, while they are significant in patients suffering from Schizophrenia and stroke (permutation test, Schizophrenia:p = 5.67e-3, depressionp = 0.332, strokep = 0.022.

The presented result is again not in agreement with the state hypothesis but can be consistent with the trait hypothesis. Although the analysis of specific diseases is not extensive and a complete list of conditions in the datasets is unknown, the presented results show that underestimation in pathological recordings can be consistently observed across different conditions. We were unable to find contradicting effects, that is, overestimation of CA. Please note that the condition labels were not provided as part of the datasets but were manually extracted from medical reports with a keyword search (seeSection 2.4.5) and that comorbidities can exist.

### 3.6 Finding 5: brain age gap is unaffected by change in pathology status in TNPP and TPNP

The top panel ofFigure 11presents brain age gaps of non-pathological versus pathological subjects (center) in the novel dataset TNPP, whose average gaps (left) are not statistically significantly different (permutation test,p = 0.825) and where the brain age gap biomarker (right) is not indicative of pathology (permutation test,p = 8.6e-2). The bottom panel ofFigure 11presents brain age gaps of non-pathological versus pathological subjects (center) in the novel dataset TPNP, whose average gaps (left) are not statistically significantly different (permutation test,p = 0.43) and where the brain age gap biomarker (right) is not indicative of pathology (permutation test,p = 0.483). For a comparison of BA and CA, please refer toFigure S14.

**Fig. 11.**
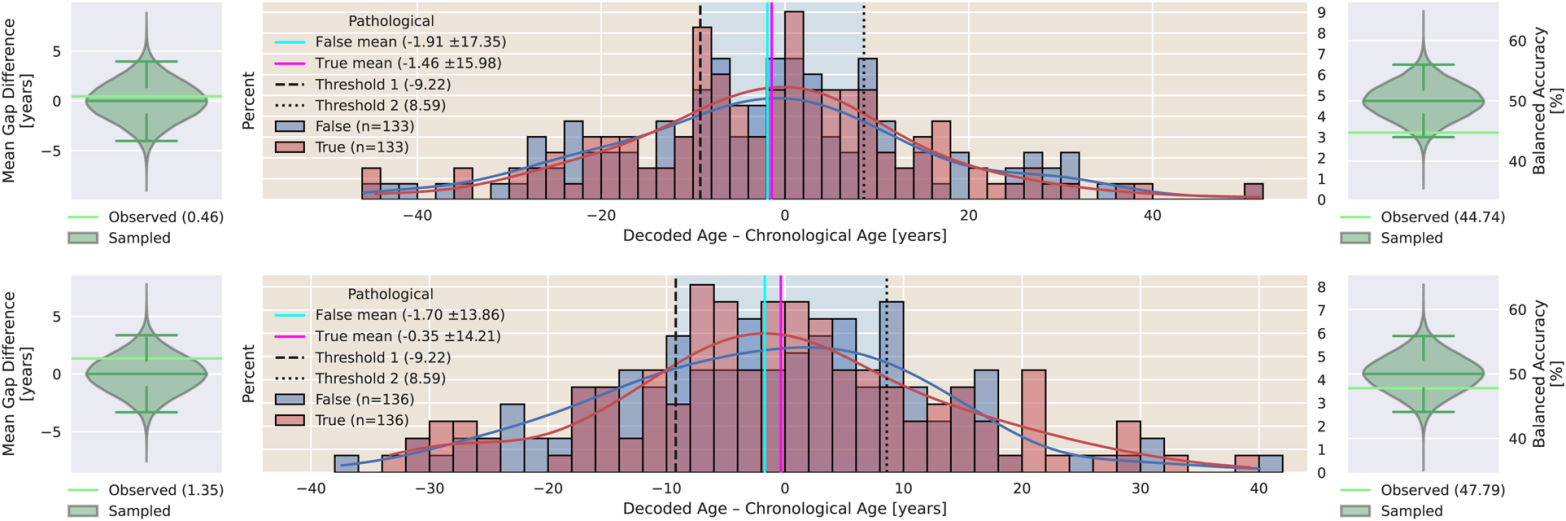
Histogram of average brain age gaps of non-pathological and pathological recordings per subject in TNPP (top) and TPNP (bottom) with permutation tests of average gap differences (left) and pathology proxy analysis (right). The cyan and magenta lines represent the average brain age gap of non-pathological and pathological subjects, respectively. Blue background signifies non-pathological classification and orange background signifies pathological classification based on thresholds found in CV presented as dashed lines. Light green lines represent actual observations whereas violins represent sampled distributions with indicators of median, 2.5th, and 97.5th percentile in darker green. The differences of average non-pathological and pathological gaps are not significant (permutation test, TNPP:p = 0.825, TPNP:p = 0.43). The application of the pathology proxy thresholds found in CV does not yield BACC scores significantly better than chance level (permutation test, TNPP:p = 0.086, TPNP:p = 0.483).

The presented finding is not in agreement with the state hypothesis as we do not find a lager brain age gap in pathological subjects and as the brain age gap is not indicative of pathology neither in TNPP nor in TPNP. However, the finding supports the trait hypothesis as we do not find significant effects on the brain age gap by acquisition or recovery of EEG pathology.

### 3.7 Finding 6: brain age change rate is identical in RNP compared to RP, and in the “Moment of Transition” in TNPP compared to TPNP

The left panel ofFigure 12presents the brain age change rate in non-pathological versus pathological subjects in RNP versus RP. Whereas the distributions of brain age change rates are significantly different (KS test,p = 3e-3), there is no evidence that the brain age change rates in RNP are significantly higher or lower compared to RP (WMW test,p=0.34; Brunner-Munzel test,p=0.27). The right panel ofFigure 12presents the brain age change rate of subjects in TNPP versus TPNP in the “moment of transition”. The distributions of TNPP and TPNP are actually the same (KS test,p=0.40), and there is no evidence that the brain age change rates in the “moment of transition” in TNPP are significantly higher or lower compared to TPNP (WMW test,p=0.13; Brunner-Munzel test,p=0.13).

**Fig. 12.**
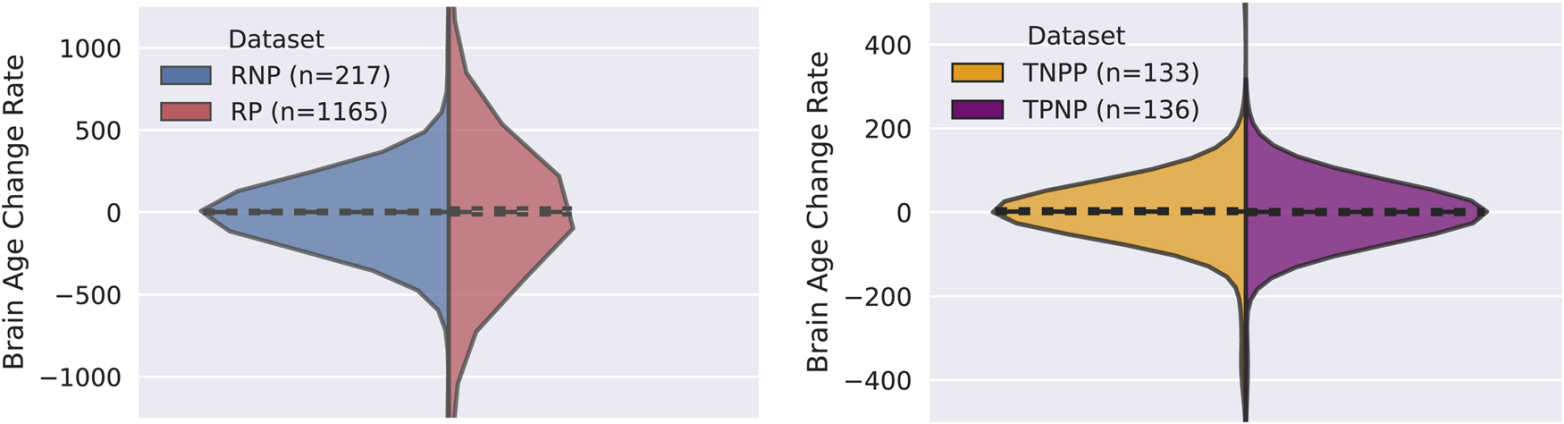
(Left) Brain age change rate in RNP versus RP. (Right) Brain age change rate in the “moment of transition” of EEG pathology label in TNPP versus TPNP. While the distributions of RNP and RP are significantly different (KS test,p=0.003), the distributions of TNPP and TPNP are actually the same (KS test,p=0.495). There is neither evidence for a higher or lower brain age change rate in RNP versus RP (WMW test,p=0.34; Brunner-Munzel test,p=0.27) nor in TNPP versus TPNP (WMW test,p=0.13; Brunner-Munzel test,p=0.13).

The presented finding is not in alignment with the state hypothesis and supports the trait hypothesis, since there is no evidence of increased or decreased brain age change rate induced by EEG pathology acquisition or recovery.

### 3.8 Finding 7: higher occipital beta activity relates to younger brain age prediction in TUAB

**Fig. 13.**
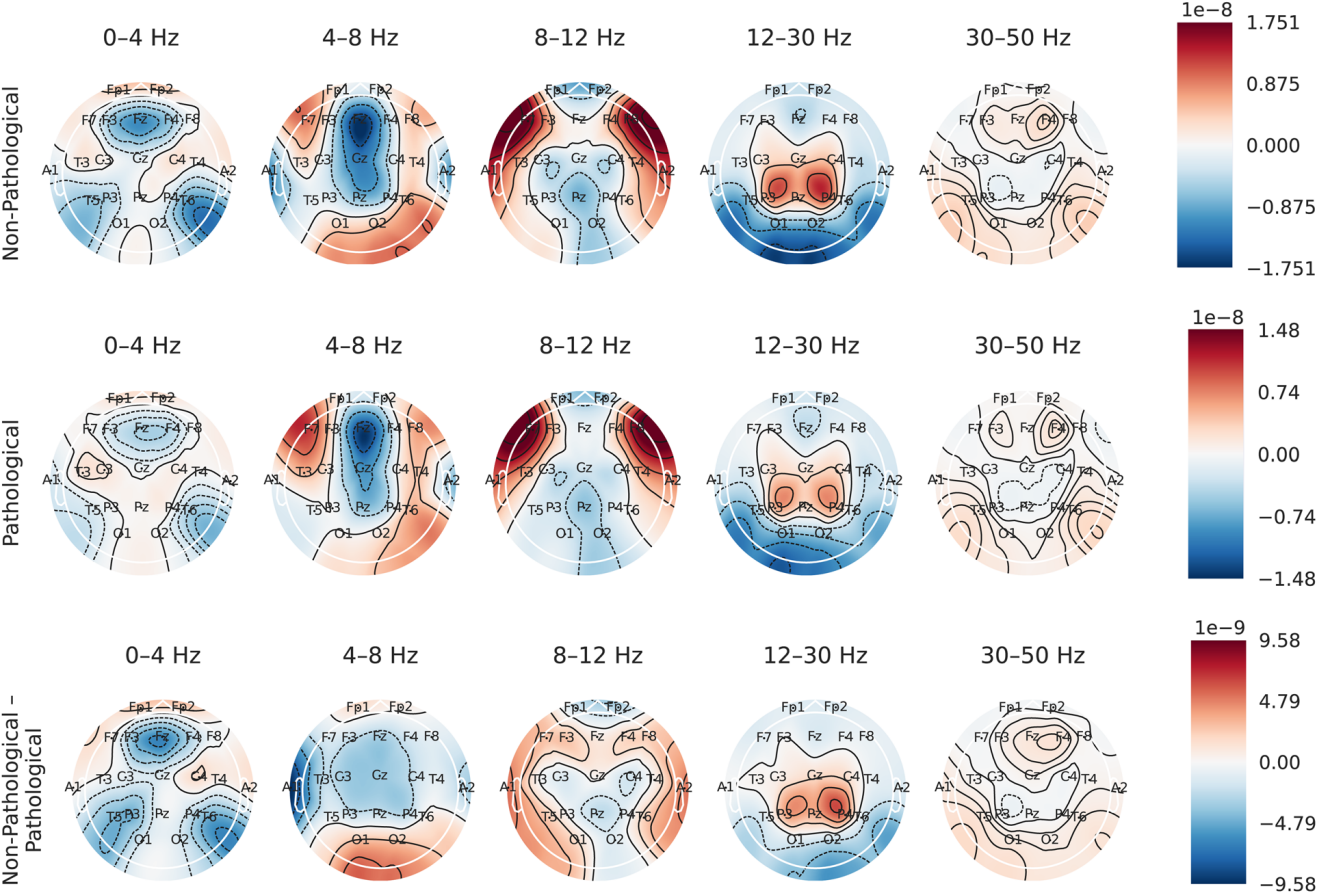
Amplitude gradient analysis with respect to different frequency bands on non-pathological (top), pathological (center) and difference of non-pathological to pathological subjects (bottom) of TUAB after FE. Negative values imply lower brain age prediction, while positive values imply higher brain age prediction. Prominent patterns across all frequency bands can be observed. In the delta frequency band, one can see three hotspots for negative gradients at frontocentral (F3, Fz, F4) and temporoparietal (T5, T6) electrode locations. In the theta frequency band, there are negative gradients at frontocentral (Fz) and vertex (Cz) electrode locations, whereas there are positive gradients at occipital (O1, O2) and frontotemporal (F7, F8) electrode locations. The latter are considerably bigger in the alpha frequency range where there are also negative gradients at frontocentral (Fp1, Fp2), parietal (T5, T6), and occipital (O1, O2) electrode locations. In the beta frequency band, one can observe positive gradients at central (C3, Cz, C4) and parietal (P3, Pz, P4) electrode locations and surrounding negative gradients are highest at occipital (O1, O2) and temporoparietal (T5, T6) electrode locations. In the gamma band, the lowest magnitude of gradients can be observed with an inverted delta pattern (positive gradients at frontocentral (F3, Fz, F4) and temporoparietal (T5, T6) electrode locations).

Figure 13presents topographical maps of gradients with the respect to the input amplitudes and shows strong and clear patterns across all frequency ranges. (This analysis goes by the name saliency maps in deep learning (Adebayo et al., 2018;Simonyan et al., 2013).) The largest absolute values can be observed in the theta, alpha, and beta frequency bands. Consequently, the network focuses less on delta and gamma bands to decode the age of subjects. A focus on the occipital brain region in the alpha band, which is one of the most prominent EEG features in healthy subjects, cannot be observed. This area is rather relevant in the beta frequency band. Note that interpretation of EEG signals is different for awake and asleep EEG. However, in the clinical setting presented here, patients should not have fallen asleep. For more information regarding brain age estimation from EEG where patients are not in an awake state, please refer toBanville et al. (2023)andSabbagh et al. (2023).

While the saliency maps clearly show the spatial and spectral importance of signals to the model, their interpretation is fairly limited. Despite the correlation of higher occipital beta power with younger brain age prediction and higher frontotemporal alpha power with older brain age prediction, one cannot conclude that these effects cause a brain to be younger. These patterns were most informative for the model to discriminate the given data. Observations might be different for other models and datasets.

## 4 Discussion

Our study found state-of-the-art EEG brain age decoding results on NP subjects of TUAB. Furthermore, our study found significant differences in brain age gap of NP compared to pathological (P) subjects both in datasets with a single recording per subject (TUAB) and datasets with multiple recordings per subject (RNP and RP). The usage of the brain age gap as a biomarker of EEG pathology yielded balanced accuracies significantly better than chance level in these datasets; however, it lacks a classification simply based on an age threshold and compared to the direct EEG pathology decoding results presented in the literature. We could neither find significant differences in brain age gap in subjects with EEG pathology status transition in either direction (NP to P or P to NP), nor when comparing the brain age gap over time nor in the “moment of transition”. Interestingly, occipital EEG activity in the beta band (12–30 Hz) appeared to be most informative for our model. To the best of our knowledge, our study is the first to investigate the potential of the brain age gap as a predictive biomarker of general EEG pathology. Furthermore, our study introduced the novel datasets RNP, RP, TNPP, and TPNP as derivatives of TUEG which enabled us to analyze the brain age gap with respect to potentially underlying mechanisms of state and trait for the very first time.

### 4.1 Comparison of brain age decoding performance

Our EEG brain age decoder achieved state-of-the-art performance of 6.6 years MAE and 0.73$R^{2}$score on non-pathological subjects of TUAB, a subset of the largest resource of clinical EEG (Section 3.2). Although our model compared well to other established models on multiple datasets via the M/EEG brain age benchmark (Section 3.2.2), we observed differences in decoding performance compared to the results listed in the original paper (Engemann et al., 2022). We hypothesize that these differences may arise from factors beyond our control, such as data releases, software and library updates, or varying seeds. Despite these differences, our training is stable; the model reaches loss convergence in CV (Fig. S10) and FE (Fig. S12) and outperforms the other contenders in three out of four cases, demonstrating its robustness.

In general, we found it difficult to compare our results to those presented in the literature, even though similar works using EEG data of non-pathological subjects exist that additionally report the MAE score. While this metric may work well for in-dataset comparisons, we find it inadequate for cross-dataset comparisons as it does not consider the true age of subjects. The same absolute error may have a greater impact on a younger patient compared to an older patient (consider 6 years MAE for a 6 year old compared to a 60 year old). Furthermore, comparisons through the MAE metric seem absurd if a brain age gap is expected, since it automatically increases the MAE even if the BA is correctly decoded. Obvious alternatives like the$R^{2}$score or an absolute percentage error (APE) suffer from other limiting factors, for example, APE encourages an underestimation if optimizing for low percentage error (McKenzie, 2011), for example, target 1, prediction 2, MAE 1, APE 100 versus target 3, prediction 2, MAE 1, and APE 33. Finding a reliable way to compare results, especially in settings where a brain age gap is expected, remains an open challenge for the brain age decoding community. For more insight on this topic, please refer tode Lange et al. (2022).

### 4.2 Brain age decoding performance in EEG compared to MRI

Despite their good performance, our models are unable to match performance of approaches based on MRI data, which achieve results around or below 3 years MAE (Smith et al., 2019,^2020^;Vidal-Pineiro et al., 2021). Based on our observations and existing literature, it can be inferred that MRI facilitates a more accurate estimation of brain age as there is no clear explanation for a systematic overestimation of brain age in EEG decoding studies compared to MRI studies. If one assumes that MRI accurately reflects the “true” age of the brain, then EEG approaches may exhibit lower precision, leading to larger errors and an increased discrepancy in brain age estimates. Further research is required to substantiate this hypothesis. One potential methodology involves the development of a combined MRI and EEG dataset to train and optimize state-of-the-art models from both domains. The resulting discrepancies in brain age estimates could then be compared to determine if they are indeed smaller for MRI compared to EEG. Additionally, the presence of individual-level correlations can be investigated. While EEG may not provide as precise an estimation of brain age as MRI, relative differences may still be preserved, such that its predictive value as a proxy measure for pathology persists.

### 4.3 Brain age gap as a predictive biomarker of EEG pathology

Despite removal of model bias (Section 3.2.1), we found an underestimation of CA of the non-pathological population (Section 3.2) as well as of the pathological population (Section 3.3), where the latter is more extreme (Section 3.3.1). These results were unexpected given the review of related literature (Franke, 2013;Sun et al., 2019;Ye et al., 2020). There, an increased brain age in pathological subjects is commonly reported and the state brain age hypothesis is predominant. Our findings are in disagreement with the state hypothesis.

Furthermore, we found that an application of the brain age gap as a biomarker of EEG pathology does not yield classification results on the same level as direct EEG pathology decoding (Gemein et al., 2020;Lopez de Diego, 2017;Roy et al., 2019;Van Leeuwen et al., 2019) (Sections3.4.1,3.4.2,3.5,3.6). However, we argue that the non-pathological population might not serve well as a control group in this setting. This is because people came to the hospital for a reason, presumably suffering from a condition that prompted an EEG examination. In the RNP dataset, people even came to the hospital repeatedly, indicating the presence of a medical condition. Pathological subjects were examined only slightly (0.47 times) more often on average compared to non-pathological subjects (Section 3.1.2). Still, since on average there are several months between follow-up EEG examinations in all datasets (Section 3.1.2), we assume that the RNP population was not excessively misclassified. A follow-up analysis of several conditions within RNP and RP show the same patterns as observed in the general populations (Section 3.5). By all means, these results are again in disagreement with the state hypothesis, since, as before, it implies higher brain age in pathological subjects. Conversely, a larger brain age gap should be indicative of pathology.

### 4.4 State vs. trait brain age

We did not find a significant difference in brain age change rate in the “moment of transition” in TNPP compared to TPNP (Section 3.7). (Note that the “moment of transition” actually refers to the time interval between subsequent EEG assessments where a label transition occurred and not to the actual moment of change of non-pathological to pathological brain activity, as this moment is unknown and likely fluent.) It is possible that the brain age increases or decreases significantly compared to prior label transition after some unknown time interval. An interesting direction for future research would be to investigate this time interval. The hypothesis could be investigated by curating additional data of subjects with repeated assessments and a selection of recordings based on short to long intervals between label transition and follow-up examinations. Our finding is not only in disagreement with the state hypothesis, but it additionally supports the trait hypothesis which implies no change through environmental factors or diseases acquisition and recovery.

### 4.5 Activity patterns of the aging brain

Our analysis of amplitude gradients (Section 3.8) revealed symmetric patterns across all frequency ranges under investigation. The human EEG has a history of close to 100 years (Haas, 2003). During this time, many researchers have investigated its properties regarding non-pathological and pathological as well as elderly and young subjects. For example,Purdon et al. (2015)have reported decreasing power in elderly compared to young subjects. One interesting line of further research would involve the application of EEG quantification methods to the presented datasets (Section 2.1.2) and a comparison to the presented gradient maps (Section 3.8). Main interests include the differences between non-pathological and pathological EEG and age-related signal alteration. For a review of literature that analyzed EEG frequencies in healthy elderly that could serve as a starting point for future analyses, please refer toMizukami and Katada (2017).

### 4.6 Limitations

We consider the presented model together with the novel datasets (Section 3.1.1) sampled from TUEG the best possible foundation to date to investigate the EEG state and trait brain age hypotheses. However, there are some limiting factors, such as an unreliability both of subject age as well as of labels indicating pathology. As discussed in our previous work (Gemein et al., 2020), pathology labels in EEG diagnostics suffer from low inter-rate agreement (Gaspard et al., 2014). Furthermore, pathology labels were only provided for the TUAB subset of TUEG by TUH. The pathology labels of the remaining data were generated through an automatic routine (Kiessner et al., 2023). While this routine yielded a conformity of 99% when applied to TUAB as a sanity check, the labels generated for RNP, RP, TNPP, and TPNP for this study were not manually reviewed. Subject age, on the contrary, should be a more reliable target as birth date and age of subjects are typically well documented. However, in our age source analysis, we found unresolved age mismatches (Supplement Section A.1) that could indicate severe mapping issues between EEG files and medical text reports within TUEG. Furthermore, the application of exclusion criteria, which we consider essential to better align with the TUAB dataset, resulted in a substantial decrease in recording and patient numbers (Section 2.1.3). Despite this outstanding resource of clinical EEG, small sample sizes are a limiting factor especially in TNPP, TPNP, and in the analysis of individual conditions from RNP and RP (Section 3.5).

## 5 Conclusion and Outlook

The fundamental interpretation of biological versus chronological brain age has recently been disputed byVidal-Pineiro et al. (2021). Based on an analysis of MRI data, the authors concluded that the difference between BA and CA is a rather stable individual trait across a lifetime, and not subject to substantial changes in response to events such as the occurrence of severe neurological disorders. In this study, we tested these state versus trait hypotheses of brain-age-dynamics using a different, important, and widely used modality for non-invasive assessment of the human brain: clinical EEG recordings. We found no evidence supporting the state brain age hypothesis and made observations to the contrary. In other words, our findings lend support to the trait hypothesis of brain-age-dynamics. Thus, our study provides findings complementary to previous MRI-based observations. However, neither our findings nor these previous findings rule out the possibility that in addition to predominant trait-like brain-age-features, more subtle brain-age-related signal components, and/or putative signal components which might not be picked up by the machine learning methods used, might follow a variable, brain-state-like pattern. Therefore, further research on brain age in different imaging modalities is required. These works could involve the decomposition through independent component analysis or a more detailed analysis of learned representations. Previous works have shown that neural networks can also be sensitive to signal phase in addition to signal power (Hammer et al., 2022;Hartmann et al., 2018). It would be interesting to perform similar analyses in the context of brain aging to better understand underlying dynamics.

In this study, we leveraged the TUEG Corpus as the largest available clinical EEG dataset. However, this dataset was not curated specifically for research into the nature of brain age and consequently, limitations exist in terms of provided labels and criteria for subject inclusion and exclusion. For more nuanced investigations of the nature of brain age, an expanded dataset featuring a larger number of subjects with repeated EEG examinations and a larger number of EEG sessions per subject would be highly beneficial. This should ideally include non-binary pathology scores compiled by multiple reliable experts, along with accurate age information that maintains anonymity.

Another interesting line of research would involve the disentanglement of the general population of EEG pathology and therefore an investigation of disease-specific brain-aging effects. An alternative dataset that could be fostered for disease-specific investigations could be the TDBRAIN dataset (van Dijk et al., 2022). The dataset includes EEG recordings of patients with diverse conditions, for example, major depression disorder, subjective memory complaints, attention deficit/hyperactivity disorder, Parkinson’s disease, insomnia, tinnitus, and others. The number of healthy subjects included in the dataset, however, is low with just 47, which complicates the training of a “normative brain age model” to begin with. It could hence be required to perform transfer learning, that is, from TUAB to TDBRAIN. Investigations on other datasets could offer deeper insights into brain aging and shed more light on the state versus trait components (and also possibly their interplay). Additionally, they could show possibly contradicting effects of specific diseases and disorders on brain aging.

## Ethical Approval

No subjects were recruited or tested in experiments for this study. Data acquisition was performed by the Temple University Hospital, Philadelphia, Pennsylvania, United States of America.

## Data and Code Availability

The TUH Abnormal EEG Corpus (Lopez de Diego, 2017) used to train the models for our study is a subset of the TUH EEG Corpus (Obeid & Picone, 2016). Both data sets are publicly available for download upon registration athttps://isip.piconepress.com/projects/tuh_eeg/. Additionally, we created several subsets of the TUH EEG Corpus. We provide all required details to recreate these datasets. Code specific to the experiments performed for the presented study as well as results were uploaded to github.com/gemeinl/eeg-brain-age.

## Author Contributions

L.A.W.G: Conceptualization, Formal analysis, Investigation, Methodology, Software, Validation, Visualization, Writing—original draft, and Writing—review & editing. R.T.S.: Conceptualization, Methodology, Supervision, Validation, and Writing—review & editing. J.B.: Project administration, Supervision. T.B.: Conceptualization, Methodology, Funding acquisition, Project administration, Resources, Supervision, and Writing—review & editing

## Funding

This work was partly supported by AI-Trust (Baden-Württemberg Foundation), Renormalized Flows (BMBF project 01IS19077C), and AI-Cog (Deutsche Forschungsgemeinschaft (DFG) project BA 4695/4-1). We acknowledge support by the Open Access Publication Fund of the University of Freiburg.

## Declaration of Competing Interest

The authors declare no competing financial interests.

## Supplementary Materials

Supplementary material for this article is available with the online version here:https://doi.org/10.1162/imag_a_00210.

## Supplementary Material

Supplementary Material

## Footnotes

1 Available for download athttps://braindecode.org

2 Available for download athttps://mne.tools

3 Available for download athttps://github.com/skorch-dev/skorch

4 Available for download athttps://scikit-learn.org/stable/

5 Available for download athttps://pytorch.org/get-started/locally/
