# Supplementary material for: Brain age revisited: Investigating the state vs. trait hypotheses of EEG-derived brain-age dynamics with deep learning
Source: Imaging Neurosci (Camb). 2024 Jul 8;2:imag-2-00210. doi: 10.1162/imag_a_00210 (PMC12301897; doi:10.1162/imag_a_00210)
Supplement: Supplementary Material [file imag_a_00210-supp.pdf]

A Supplement

A.1 Comparison of Sources of Age Information

To extract age information from TUEG, we found multiple possible methods.

I) **Header age:** Age can be parsen from the EDF recording file header where bytes 8 to 88 of the file are dedicated to patient information [Kemp et al. (1992)] and age is usually presented as "Age: XX".

II) **Report age:** Age can be parsed from the medical text reports, which typically start with phrases like "This is a XX-year old / XX y.o." or variations thereof, however, some medical reports did not follow this convention.

III) **Date age:** Age can be computed based on the anonymized patient birth date and the date of the recording. The birth date is also included in the patient information section of the EDF header, while the recording date can be obtained from measurement information contained in the EDF file.

We compared all three extraction methods in Figures S1 and S2.

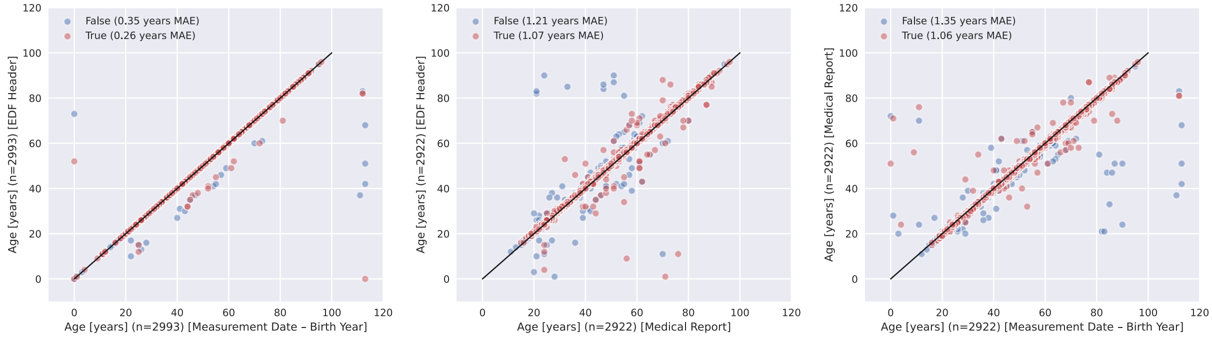

Figure S1: Different sources of age information in TUAB: left: date vs. header, center: report vs. header, right: date vs. report. Markers represent individual recordings. Orientation along the diagonal signifies agreement of age in both sources. Ages of the different sources do not match in all cases. The highest consensus can be found in date and header age (left panel), although there is a systematic error of 10 years for some recordings in date age. Matching report age with the other sources reveals a diffuse pattern of higher/lower age in one of the sources.

From the comparison plots of age sources, we observed some interesting patterns. For example, in the comparison of date and header age in TUAB (left panel of Figure S1) we noticed a systematically higher

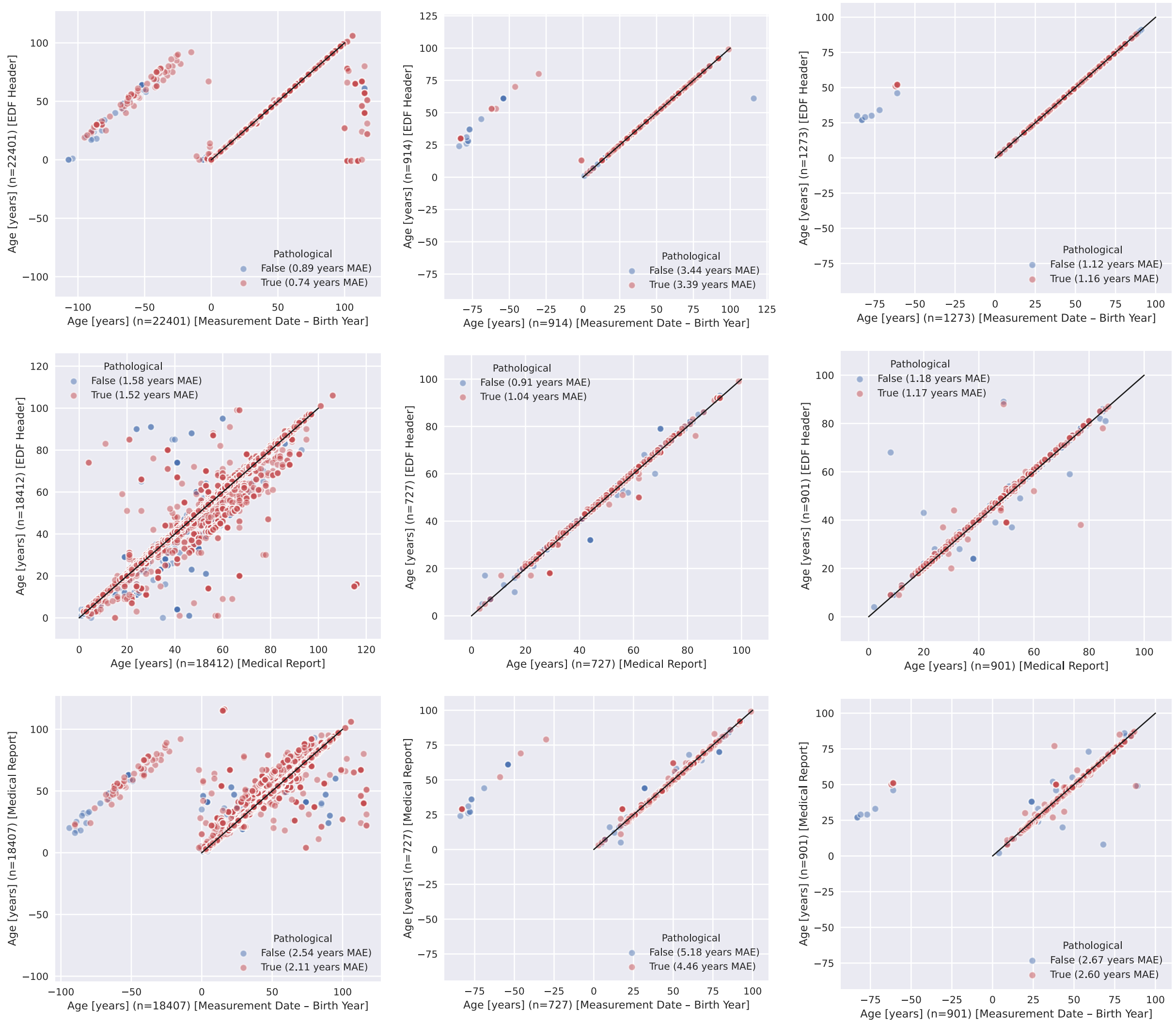

Figure S2: Different sources of age information (from top to bottom: date vs. header, report vs. header, date vs. report) in RNP vs. RP (left), TNPP (center), and TPNP (right). Markers represent individual recordings. Orientation along the diagonal signifies agreement of age in both sources. Ages of the different sources do not match in all cases. There is a systematic error of -100 years for some recordings in date age. Matching report age with other sources reveals a diffuse pattern of higher/lower age in one of the sources.

age of ten years for some recordings. When comparing report age (central and right panel of Figures S1) we observed a diffuse pattern of over- and underestimation for some recordings. The reason for these differences is unclear, but one possible explanation could be a misalignment of EDF files and medical reports within TUEG. According to the mean absolute error (MAE) metric (Section 2.3), the agreement of age is best between date and header age.

We made similar observations when comparing age sources in our datasets with repeated examinations (Figure S2). Matching date age revealed a systematic age difference of -100 years for some recordings, e.g. top left panel of Figure S2, while matching report age revealed a diffuse pattern as in TUAB. According to the MAE metric, the agreement of age is best between date and header age for RNP, RP, and TPNP and between report and header age in TNPP.

Since these comparisons revealed a substantial amount of label noise, we use the presented age sources to reject recordings (Section 2.1.3). We considered age information extracted from the EDF header to be most reliable, as it does not require matching files (medical report and EDF) or computation (date from EDF measurement info and birth year extracted from EDF header), and chose it as our decoding target. However, the header age might suffer from anonymization imprecision of one year due to the omission of day and month of birth date. For the rest of this study, when we refer to (chronological) subject age or age labels, we refer to the age extracted from EDF headers.

A.2 Inclusion and exclusion

Our inclusions and exclusion rules (Section 2.1.3) had effect on the number of patients and recordings. Details are provided in Table S1. The rightmost column contains the values of the datasets as used for the main results of this work.

A.3 Recording Artifacts in TUAB

As in our previous work [Gemein et al. (2020)], we removed outliers at the start of EEG recordings during preprocessing (Section 2.2.1). To substantiate this decision, Figure S3 shows an analysis of the first three minutes of recordings in TUAB. One can see that the number of outliers $\pm 800\mu V$ is highest in the first

	#recordings / #subjects	#recordings / #subjects	#recordings / #subjects
	**duration $\geq$ 2 min**	**duration $\geq$ 15 min**	**duration $\geq$ 15 min & reliable age**
**TUAB**	2.993 / 2.329	2993 / 2.329	**2647 / 2.159**
**RNP**	4.068 / 956	933 / 372	**621 / 245**
**RP**	18.338 / 2.892	5.112 / 1.672	**3.963 / 1.321**
**TNPP**	914 / 195	431 / 170	**330 / 133**
**TPNP**	1.273 / 242	563 / 216	**347 / 136**

Table S1: Number of recordings / subjects of datasets used for this study when changing inclusion criteria (i.e. longer recordings and reliable age information across the different sources). The rightmost column contains the values of the datasets as used for this work.

minute and decreases in the following minutes. We assume that some readjustment of the electrode cap or finding a comfortable seating position takes place in the first minute and hence causes these outliers.

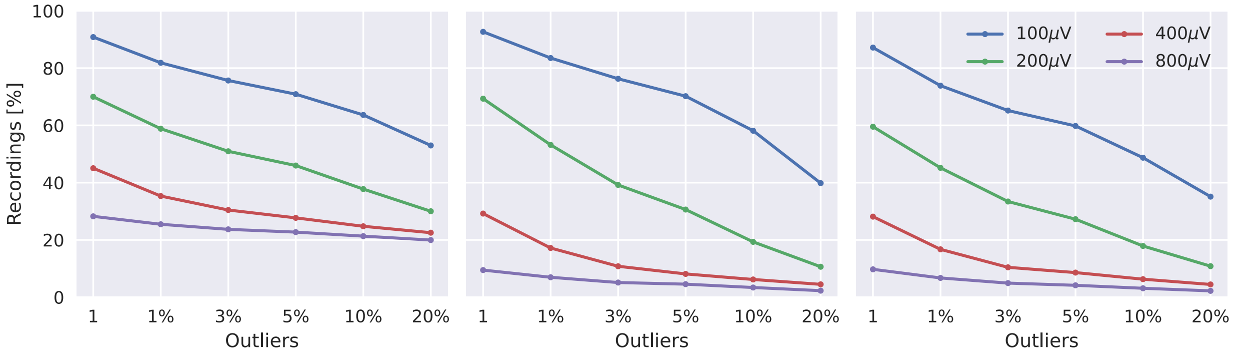

Figure S3: Unphysiologically high values at the start (first three minutes) of recordings in TUAB. From left to right: Outliers in the first, second, and third minute of recordings. In the first minute, 20% of values are outliers in 20% of the recordings. There is a decrease in outliers above all voltage thresholds (100, 200, 400, and 800μV) from first over second to third minute. The decrease is most prominent in high voltages (400 and 800μV) from first to second minute.

920 ## A.4 Pipeline Comparison to M/EEG Brain Age Benchmark

The presented decoding pipeline differs from the one presented in Engemann et al. (2022). Details and reasoning can be retrieved from Table S2.

**Current study**	**Study by Engemann et al.**	**Reason**
No conversion of raw EDF files	Conversion of raw EDF files to BIDS [Pernet et al. (2019)] format	Reduced overhead and sparing resources
Reject recordings with high ($>$ 1 year) age derivation (Figure S1)	No recording rejection based on age	Assumed to be labeling errors
Discard first 60 s of each recording	No cropping of recordings	More outliers in first 60 s of recordings (Figure S3)
Resampling to 100 Hz	Resampling to 200 Hz (49 Hz low-pass afterwards)	Clinical EEG unlike to contain informative brain activity $>$ 50 Hz
Simple preprocessing rules	Application of Autoreject [Jas et al. (2017)]	ConvNets are an end-to-end method which should be able to learn brain signals as well as to ignore artifacts
5-fold cross-validation	10-fold cross-validation	Twice as many recordings in each validation set, hence better representation of the joint underlying distribution; additionally, strong reduction of runtime
Final evaluation	No final evaluation	Possibility to tune hyperparameters in CV and compute generalization error afterwards

Table S2: Differences in design of decoding pipeline of this study compared to the M/EEG Brain Age Benchmark study [Engemann et al. (2022)].

A.5 Descriptive Statistics

A.5.1 Recording Durations

Whereas recordings of TUAB were selected to have a duration of at least 15 minutes [Lopez de Diego (2017)], this does not apply to the entirety of recordings contained in TUEG and hence our novel datasets. Therefore, we show the distribution of recording duration in RNP, RP, TNPP, and TPNP in Figure S4. We introduce a minimal recording duration of two minutes as we drop the first minute of recordings during preprocessing, due to higher amount of outliers (Section A.3). To better align with TUAB, we made selections of recordings based on duration in our decoding pipeline (Section 2.1.2).

A.5.2 Non-pathological Subset of TUAB

We present an age pyramid of the non-pathological recordings in TUAB in Figure S5. The histograms show that the distribution of ages is similar in the training and final evaluation sets, which was the main reason

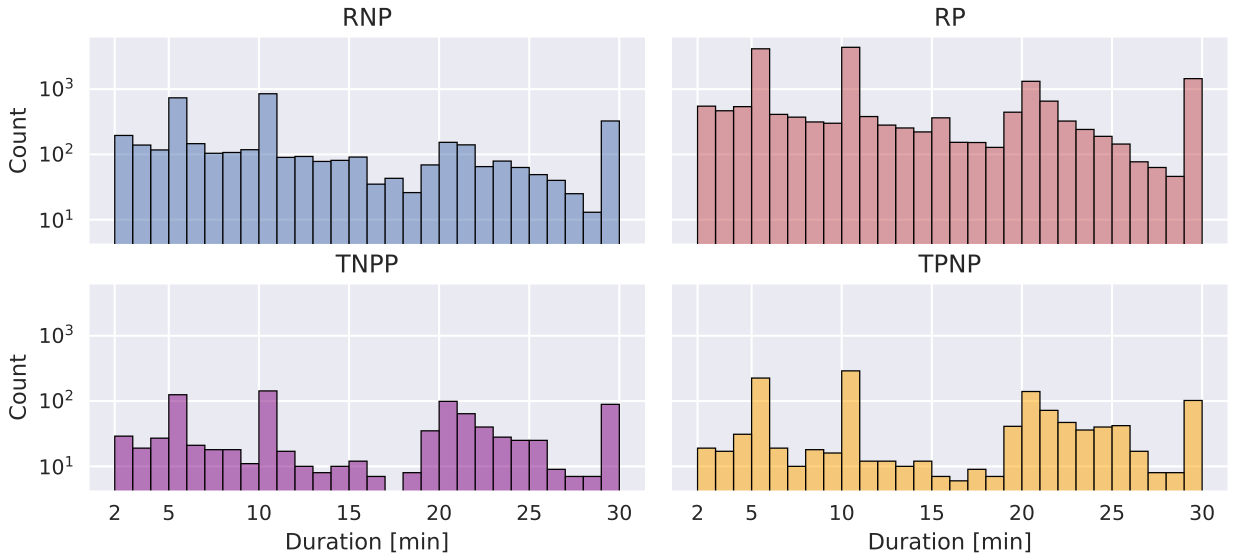

Figure S4: Durations of recordings in RNP, RP, TNPP, and TPNP. The last bin contains all recordings with a duration of 30 minutes or longer. There are prominent peaks at 5 and 10 minutes and there is a less prominent peak at 20 minutes.

we re-split the dataset. For a visualization of the entire dataset including pathological subjects, please refer to Gemein et al. (2020).

A.5.3 CV Splits

We present age histograms of our CV splits in Figure S6. For CV we can observe slight variations in age distribution, especially in the validation set. The relatively high variance regarding number of recordings is caused by the subject-wise data splitting procedure.

A.6 Hyperparameters

In Table S3 we present our model hyperparameters. Additional hyperparameter choices are listed in Table S4.

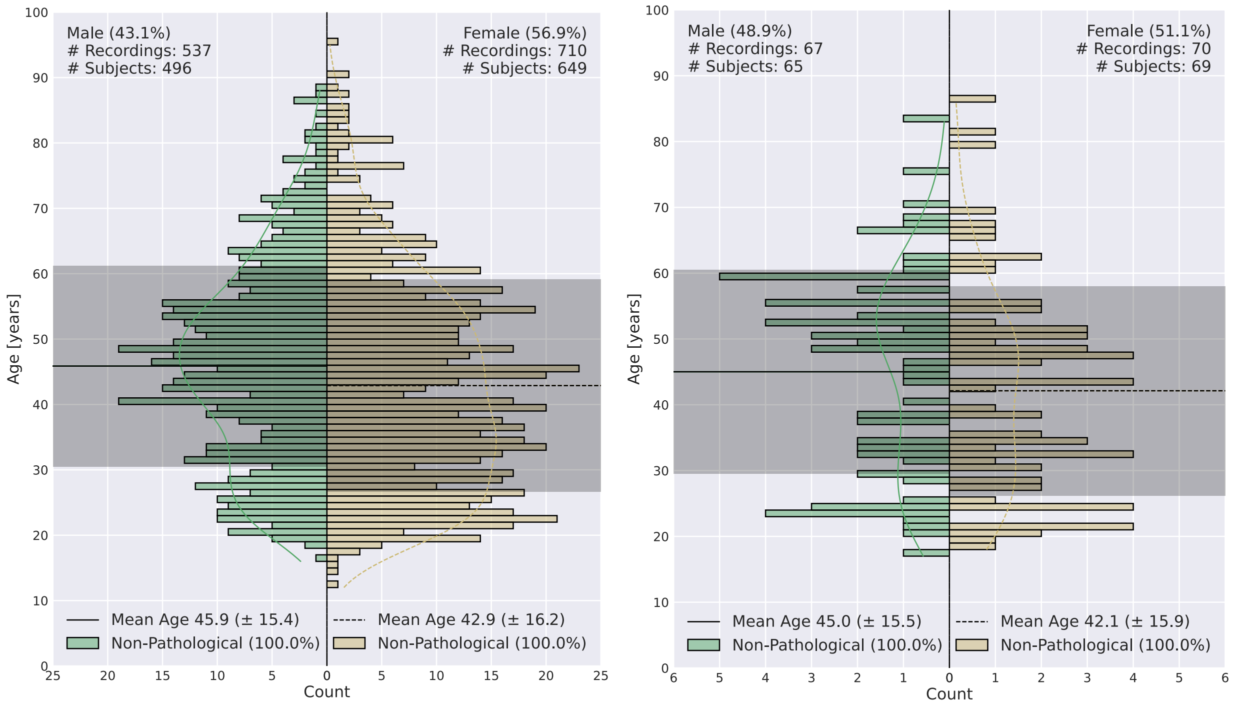

Figure S5: Left: age pyramid of training and right: final evaluation split of non-pathological recordings in TUAB. There is a higher amount of female recordings and subjects (710/649) in the training split compared to the final evaluation split (70/69). Average ages and standard deviation are similar between the data splits.

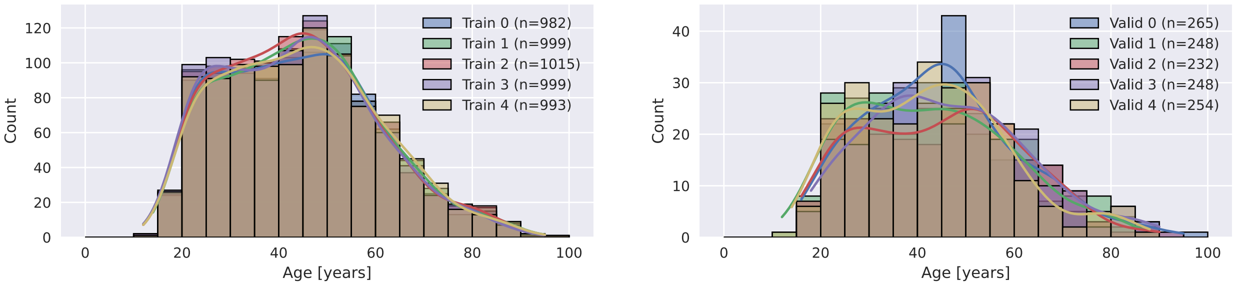

Figure S6: Left: Age distributions of non-pathological recordings in training splits of 5-fold CV. Right: Age distributions of non-pathological recordings in validation splits of 5-fold CV.

A.7 Cross-validation Results

In CV our brain age decoders achieved 6.65 years MAE on average. On pathological subjects in contrast, on which the models were not trained, the models did not reach comparable scores with 11.90 years MAE.

batch_size	128
max_epochs	35
n_chans	21
dropout_prob	0.0195875974361336
init_lr	0.0004732953501425473
weigth_decay	1.0025958447703478e-07
n_filters	53
n_blocks	5
kernel_size	9

Table S3: Model hyperparameters.

input_time_length	6000
loss	l1
channel_dropout_prob	.2

Table S4: More hyperparameters.

Figure S7 shows decoded brain ages in relation to chronological ages as well as their distributions.

We present the distributions of EEG brain age gaps of non-pathological and pathological subjects and the permutation test distributions of average gap differences in Figure S8.

Fitting a simple age threshold results in a superior BACC (Figure S9). The presented learning curves (Figure S10) show neither signs of over- nor underfitting and consistently converge close to the specified epoch maximum. Apart from one run, there is little variance between final loss values of the different runs. Apart from the convincing model fit suggested by Figures S10 and 3, we observed a prediction bias in our model.

We present the quadratic model fit to reduce the bias and the effect of its application in Figure S11.

A.8 Learning Curves in Final Evaluation

We present the learning curves of our models in FE in Figure S12. There is minor variation in final evaluation loss value, due to varying initizlization seeds.

A.9 Decoding Scores

For age regression we have computed the R^2 score in addition to the MAE score. For pathology classification based on brain age gap thresholds, we have computed the BACC score. We report all decoding scores on all

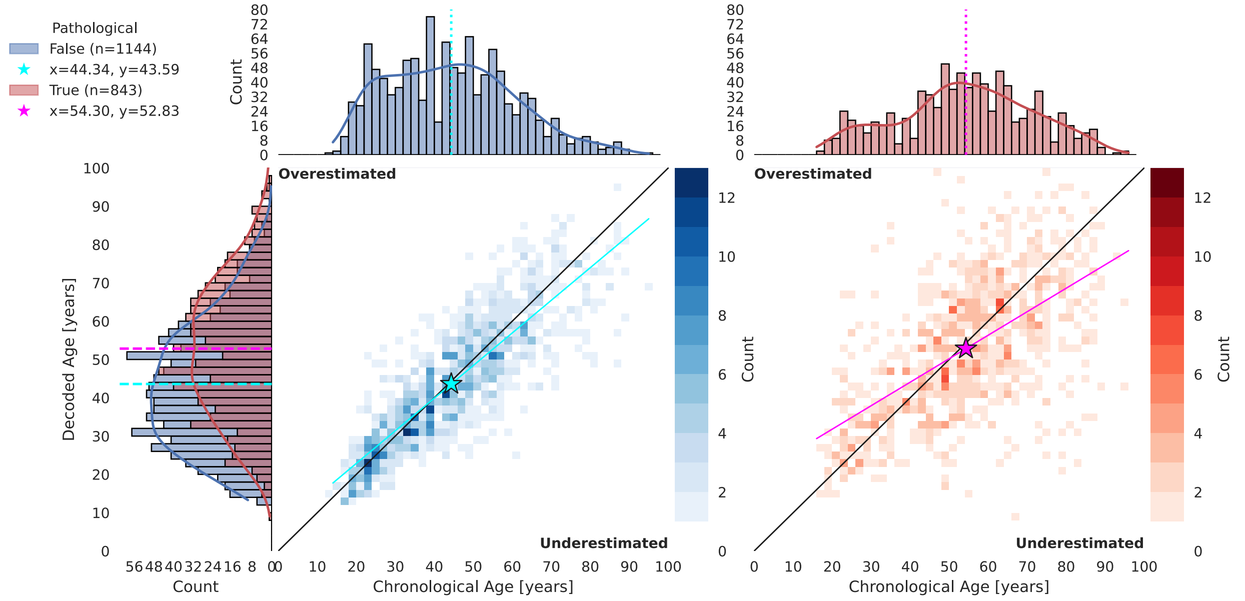

Figure S7: 2D histogram of decoded and chronological age with distributions in CV respecting the pathology status. Our models tend to overestimate younger subjects and to underestimate older subjects as very common in these decoding settings. This trend can be consistently observed on non-pathological as well as on pathological subjects through the cyan and magenta line. The effect is stronger for pathological subjects than for non-pathological subjects. The 2D histogram with average markers shown in cyan and magenta reveal a general underestimation of the non-pathological population which is even bigger for the pathological population.

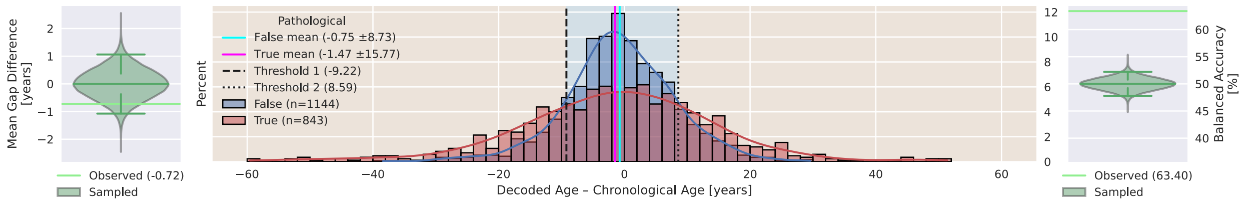

Figure S8: Left: Histogram of brain age gaps respecting the pathology status with average gaps. Right: Permutation test of average gap differences with respect to pathology status observed in CV. The observed average gaps shown in cyan and magenta are negative. The ones for pathological subjects are smaller (-1.47) than the ones for non-pathological subjects (-0.75). There is no statistical evidence that the average gap difference (-0.72) is significant. The gap distribution of brain age gaps of pathological subjects shows roughly twice the amount of variance (+-16) of the distribution of non-pathological subjects (+-8.5).

subset in Table S5.

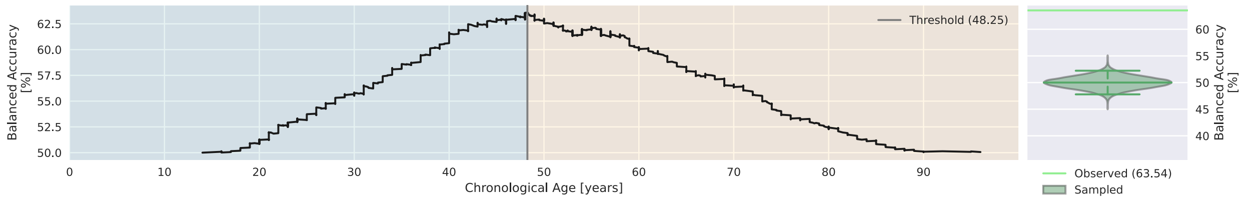

Figure S9: Pathology label assignment based on age threshold with permutation test. A threshold of 48.25 years yielded optimal BACC of 63.54%.

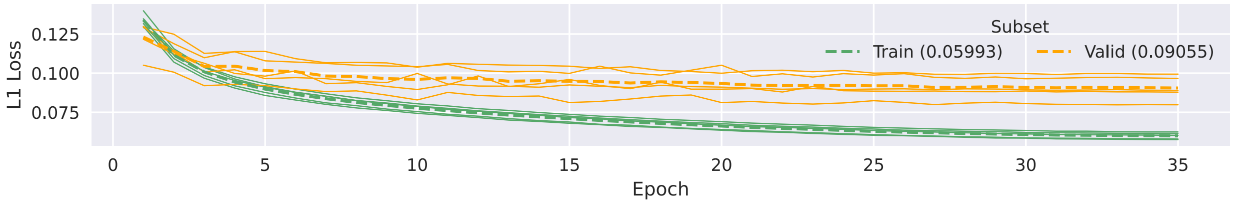

Figure S10: Loss curves are smooth and saturate well before the specified epoch maxium. There is slight variation in final validation score, due to data split.

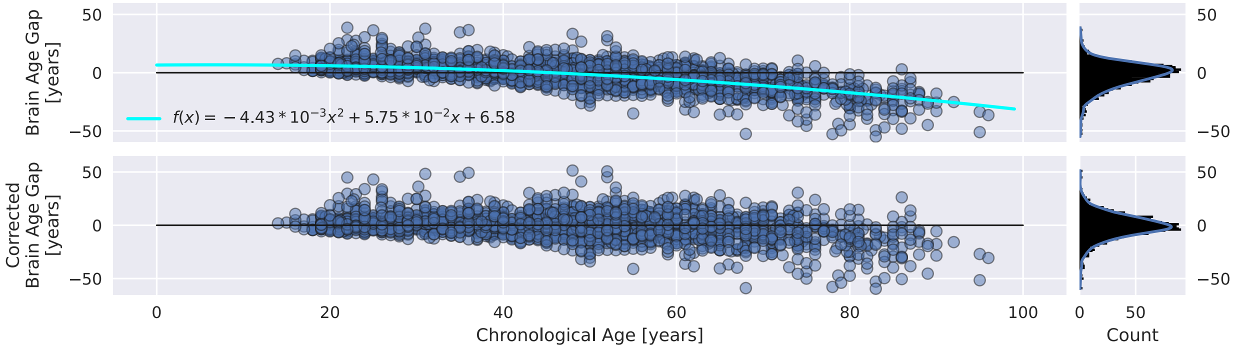

Figure S11: Presentation of fit and application of the quadratic bias model to CV data. Clearly, the gap distribution (top right) is positively skewed, not centered around zero and hence biased. The light blue curve represents the bias model and shows its fit to the data which is not aligned with the baseline. In the lower plot, one can see how the application of the quadratic bias model affects the brain age gaps. As a result, their distribution (bottom right) is symmetrically centered around zero.

A.10 Analyses on RNP, RP, TNPP, and TPNP

Below we show decoding results after FE on the new datasets RNP, RP, TNPP, and TPNP.

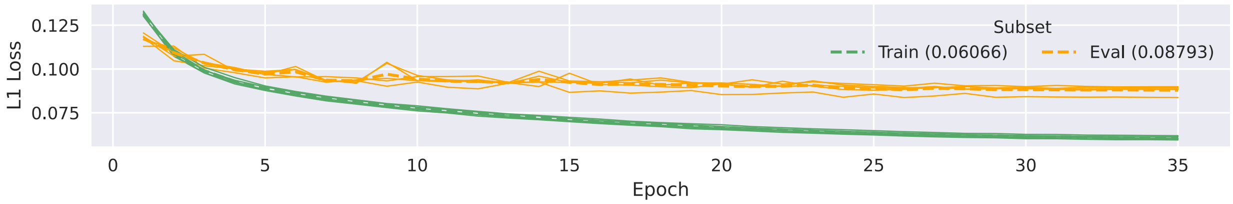

Figure S12: Learning curves of five repetitions of final evaluation. Curves are smooth and saturate well before the specified epoch maximum. Apart from one run, final evaluation loss value is similar for all runs.

	CV		FE					
Dataset	TUAB NP	TUAB P	TUAB NP	TUAB P	RNP	RP	TNPP	TPNP
MAE	6.65	11.90	6.60	12.82	9.27	14.87	12.71	11.02
R^2	0.70	0.20	0.73	0.03	-0.22	-1.63	-0.4	-0.36

Table S5: All age decoding scores on all subsets.

	CV	FE			
Dataset	TUAB	TUAB	RNP vs. RP	TNPP	TPNP
BACC	63.40	61.12	60.80	44.74	47.79

Table S6: All pathology decoding scores based on brain age gap thresholds on all subsets.

A.11 Statistical Analyses

In the table below, we detail the various statistical tests conducted as part of this study. We mainly chose permutation tests, as these tests are non-parametric and their logic intuitive. Furthermore, permutation tests do not require any specific distribution of the data tested. In addition we used a KS test to assess whether data is normally distributed as this is a condition for the Brunner-Munzel and the WMW test. The following terminology is used:

BACC: Balanced accuracy

BAG: brain age gap (BA - CA: biological age of the brain - chronological age)

NP: Non-pathological subjects

P: Pathological subjects

T: Threshold

**Test**	**Null Hypothesis**	**Variables**	**Outcome**	**Result**
Paired T-test	The means of X and Y are identical	X: BAG of NP in TUAB, Y: 0	p=0.18	3.2.1
Paired T-test	The means of X and Y are identical	X: BAG of P in TUAB, Y: 0	p=6.6e-3	3.3
Permutation test	X and Y come from the same distribution	X: BAG of NP in TUAB, Y: BAG of P in TUAB	p=1.63e-2	3.3.1
Permutation test	The BACC of the BAG biomarker is at chance level (50%)	X: BACC of BAG in TUAB < T1 or BAG in TUAB > T2 → P and T1 ≤ BAG in TUAB ≤ T2 → NP & Y: 50	p=1.32e-3	3.4.1 top
Permutation test	The age threshold BACC is at chance level (50%)	X: BACC of CA in TUAB > T → P and CA in TUAB < T → NP & Y: 50	p=1e-5	3.4.1 bottom
Permutation test	X and Y come from the same underlying distribution	X: BAG in RNP & Y: BAG in RP	p=1e-5	3.4.2 left
Permutation test	The BACC of the BAG biomarker is at chance level (50%)	X: BAG in RNP and RP < T1 or BAG in RNP and RP > T2 → P & Y: T1 ≤ BAG in RNP and RP ≤ T2 → NP	p=1e-5	3.4.2 right
Permutation test	X and Y come from the same underlying distribution	X: BAG of NP in TNPP & Y: BAG of P in TNPP	p=0.825	3.6 top left
Permutation test	The BACC of the BAG biomarker is at chance level (50%)	X: BAG in TNPP < T1 or BAG in TNPP > T2 → P & Y: T1 ≤ BAG in TNPP ≤ T2 → NP	p=8.6e-2	3.6 top right
Permutation test	X and Y come from the same underlying distribution	X: BAG of NP in TPNP & Y: BAG of P in TPNP	p=0.43	3.6 bottom left
Permutation test	The BACC of the BAG biomarker is at chance level (50%)	X: BAG in TPNP < T1 or BAG in TPNP > T2 → P & Y: T1 ≤ BAG in TPNP ≤ T2 → NP	p=0.483	3.6 bottom right
Brunner Munzel	X and Y come from the same underlying distribution	X: Change rate of BAG in RNP & Y: Change rate of BAG in RP	p=0.34 & p=0.27	3.7 left
KS Test	X and Y come from the same underlying distribution	X: Change rate of BAG of NP → P in TNPP & Y: Change rate of BAG of P → NP in TPNP	p=0.4	3.7 right
Brunner Munzel	X and Y come from the same underlying distribution	X: Change rate of BAG of NP → P in TNPP & Y: Change rate of BAG of P → NP in TPNP	p=0.13	3.7 right
WMW Test	X and Y come from the same underlying distribution	X: Change rate of BAG of NP → P in TNPP & Y: Change rate of BAG of P → NP in TPNP	p=0.13	3.7 right

Table S7: Summary of statistical tests conducted for this study.

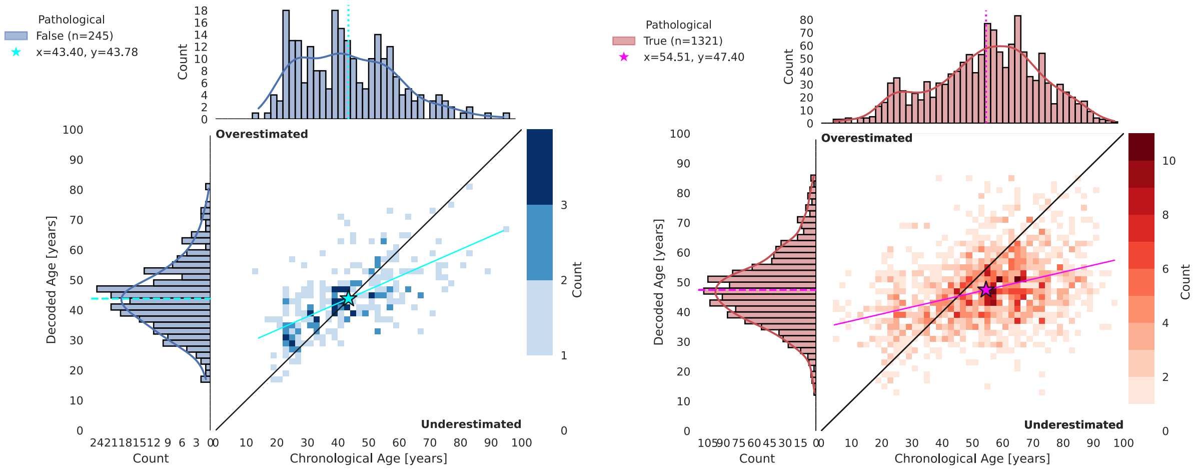

Figure S13: 2D histogram of decoded and chronological age with distributions in FE in RNP (left) and RP (right). Our models tend to overestimate younger subjects and to underestimate older subjects as very common in these decoding settings. This trend can be consistently observed on non-pathological as well as on pathological subjects through the cyan and magenta line. The effect is stronger for pathological subjects than for non-pathological subjects. The 2D histogram with average markers shown in cyan and magenta reveal a general overestimation of the non-pathological population and an underestimation of the pathological population.

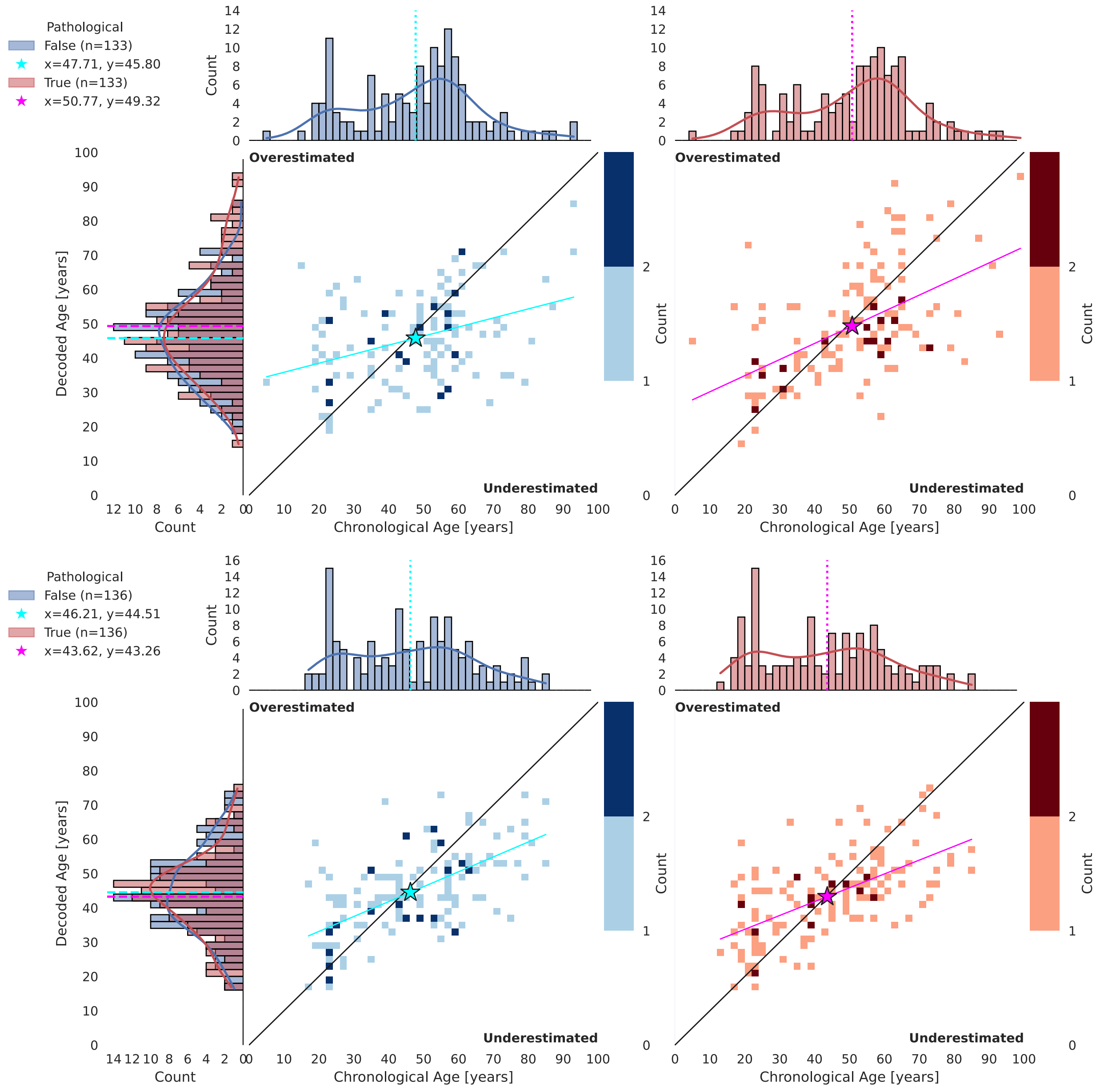

Figure S14: 2D histogram of decoded and chronological age with distributions in FE in TNPP (top) and TPNP (bottom) respecting the pathology status. Our models tend to overestimate younger subjects and to underestimate older subjects as very common in these decoding settings. This trend can be consistently observed on non-pathological as well as on pathological subjects through the cyan and magenta line. The effect is stronger for pathological subjects than for non-pathological subjects. The 2D histogram with average markers shown in cyan and magenta reveal a general underestimation of both populations.
